# Opposing Roles for Interferon Regulatory Factor-3 (IRF-3) and Type I Interferon Signaling during Plague

**DOI:** 10.1371/journal.ppat.1002817

**Published:** 2012-07-26

**Authors:** Ami A. Patel, Hanni Lee-Lewis, Jennifer Hughes-Hanks, Craig A. Lewis, Deborah M. Anderson

**Affiliations:** 1 Department of Veterinary Pathobiology, University of Missouri, Columbia, Missouri, United States of America; 2 Laboratory for Infectious Disease Research, University of Missouri, Columbia, Missouri, United States of America; 3 Starling Enterprise, LLC, Columbia, Missouri, United States of America; Tufts University School of Medicine, United States of America

## Abstract

Type I interferons (IFN-I) broadly control innate immunity and are typically transcriptionally induced by Interferon Regulatory Factors (IRFs) following stimulation of pattern recognition receptors within the cytosol of host cells. For bacterial infection, IFN-I signaling can result in widely variant responses, in some cases contributing to the pathogenesis of disease while in others contributing to host defense. In this work, we addressed the role of type I IFN during *Yersinia pestis* infection in a murine model of septicemic plague. Transcription of IFN-β was induced *in vitro* and *in vivo* and contributed to pathogenesis. Mice lacking the IFN-I receptor, *Ifnar*, were less sensitive to disease and harbored more neutrophils in the later stage of infection which correlated with protection from lethality. In contrast, IRF-3, a transcription factor commonly involved in inducing IFN-β following bacterial infection, was not necessary for IFN production but instead contributed to host defense. *In vitro*, phagocytosis of *Y. pestis* by macrophages and neutrophils was more effective in the presence of IRF-3 and was not affected by IFN-β signaling. This activity correlated with limited bacterial growth *in vivo* in the presence of IRF-3. Together the data demonstrate that IRF-3 is able to activate pathways of innate immunity against bacterial infection that extend beyond regulation of IFN-β production.

## Author Summary

Type I interferons (IFN-I) broadly stimulate innate immunity against viral, bacterial and parasitic pathogens. Many bacterial pathogens induce IFN-I through phosphorylation of Interferon Regulatory Factor 3 (IRF-3) allowing it to bind promoters containing Interferon Stimulated Response Elements (ISRE) which include IFN-β and pro-inflammatory cytokines and chemokines. Secreted IFN-β is taken up by the IFN-αβ receptor (IFNAR), triggering activation of the JAK-STAT pathway which also activates ISRE-containing genes. In this work, we have discovered a novel anti-bacterial function of IRF-3. We show that the respiratory pathogen, *Yersinia pestis*, the causative agent of plague, activates IRF-3 and the IFN-I response and that these two events cause opposite outcomes in the host. While IRF-3 is necessary for an early stage of phagocytosis, IFNAR signaling promotes the infection and may directly contribute to neutrophil depletion during infection. These results demonstrate that an IFN-independent function of IRF-3 is important to host defense against bacterial infection.

## Introduction

Type I interferons (IFN-I) are expressed by macrophages and epithelial cells as part of the first line of defense against infection, and the IFN-I receptor (IFNAR) is expressed by most cells [1]. IFN-I signaling following bacterial infection leads to the production of pro-inflammatory cytokines and chemokines and promotes apoptosis of infected cells [2]. In some cases, a pathologic role for IFN-I activation has also been described [3]. Interferon regulatory factor 3, IRF-3, is a major transcription factor that induces IFN-I following cytosolic detection of a pathogen [4]. Toll-like receptor (TLR) activation or other host pattern recognition receptors signal independent of the adaptor MyD88 to phosphorylate IRF-3 and activate IRF-3 dependent innate immune defenses [5]. Following phosphorylation, IRF-3P is found in the nucleus where it forms a complex with p300 which can act as a potent transcription factor, binding to interferon stimulated response elements (ISREs) on target genes, including *Ifnβ* [6], [7]. Secreted IFN-β binds IFNAR and signaling through STAT-1 and STAT-2 (signal transducers and activators of transcription) induces transcription of hundreds of ISRE-containing genes including many pro-inflammatory cytokines and chemokines. Further amplification of IFN-β expression occurs through an autocrine loop that requires IFNAR, IRF-3 and a second transcription factor IRF-7 and all three proteins play key roles in the expression of IFN-I [8].

Intracellular pathogens, such as *Listeria monocytogenes*, trigger activation of IRF-3 and subsequent induction of IFN-β as a result of their escape from the phagolysosome and replication within the host cytosol [9]. Subsequent IFN-β signaling in leukocytes, not necessarily infected by *L. monocytogenes*, promotes apoptosis, which is thought to result in a reduction of the number of effector cells available to defend against the infection [10], [11], [12]. Further immune suppression is caused by an IRF-3-dependent down-regulation of the IFN-γ receptor on macrophages, rendering them unresponsive to type II IFN [13]. Thus, for *Listeria*, IFN-I signaling is exploited as a virulence mechanism, allowing the bacterium to disarm the host immune system. However, other intracellular bacterial pathogens, such as *Legionella pneumophila* are successfully combated by the IFN-I response, and the replication of these bacteria appears directly affected [14], [15], [16].

Release of bacterial DNA following phagocytosis commonly activates IRF-3, leading to pro-inflammatory cytokine production necessary for neutrophil recruitment and bacterial clearance [17], [18], [19], [20], [21]. Furthermore, cytosolic activation of the AIM2 inflammasome requires IRF-3 and IFN-β which subsequently initiate caspase-1-dependent pyroptosis and the secretion of the pro-inflammatory cytokines IL-1β and IL-18 [22]. However, IFN-I signaling can also inhibit the inflammasome from being activated via NLRP3 [23]. These data demonstrate that downstream effects of IFN signaling can be dependent on the mechanism of activation.

Some bacteria, such as *Yersinia pestis,* are highly inflammatory even though they synthesize an altered lipopolysaccharide structure that poorly stimulates TLRs [24]. Deletion of *Tlr2* or *Tlr4* does not increase sensitivity or resistance to *Y. pestis* infection in mouse models, indicating that these pathways are likely not activated during the infection [24], [25], [26]. In the lungs, *Y. pestis* establishes an anti-inflammatory environment that is permissive to bacterial replication [27]. Following this initial anti-inflammatory state, robust neutrophil recruitment in response to the pathogen occurs but is ineffective [28]. Loss of CXC-chemokine signaling, a major pathway for neutrophil recruitment and activation, results in increased sensitivity to plague [29]. Though neutrophils are recruited early to infected lungs of *Cxcr2^−/−^* mice, they have reduced capacity to limit bacterial growth. Together these data suggest that wild type *Y. pestis* induces multiple pathways of neutrophil chemotaxis and is at least partially resistant to neutrophil killing. In contrast, if *Y. pestis* lack the pigmentation locus (pgm^−^), a 102 kb chromosomal deletion that attenuates virulence, CXCR2 is not required for host defense suggesting that the pgm locus may be involved in neutrophil resistance.

Infection by *Y. pestis* causes plague, a lethal disease that is characterized by rapid bacterial growth, massive pro-inflammatory responses and tissue necrosis which lead to the rapid demise of mammalian hosts including humans [28], [30], [31], [32]. Late stage disease involves vascular dissemination, rapid replication of extracellular bacteria and the development of high titer septicemia. In contrast, early infection may involve an intracellular stage, as the bacteria survive well but grow slowly inside activated macrophages [28], [33]. Perhaps due to this intracellular form, *Y. pestis* is thought to initially suppress inflammation causing an apparent biphasic inflammatory response to infection [28], [32], [34], [35]. Production of IFN-γ, normally an effective response to activate macrophages and other phagocytic cells to destroy extracellular pathogens, is initially prevented and when given as a therapeutic to mice during the first 24 hrs post-infection, the bacteria are cleared without development of disease [36], [37], [38].

Recently, a related pathogen, *Y. pseudotuberculosis*, was shown to induce MyD88-independent type I IFN and NF-κB activation following infection of macrophages *in vitro* [39]. Infection resulted in increased transcription of the IFN regulatory factor, IRF-1, as well as many IFN-responsive genes. Activation of type I IFN and NF-κB were not caused by intracellular bacteria, as they could not be prevented by cytochalasin D. Instead, the bacterial type III secretion system, but not its secreted effector proteins, was shown to be necessary for induction of both IFN-I and NF-κB responses leading to the hypothesis that the host may sense insertion of the type III translocation pore to activate innate immunity. However, these studies left unresolved the role of the IFN-I response in *Yersinia* pathogenesis.

In this work, we investigated the role of IFN-I during pulmonary infection of mice by pgm^−^ *Y. pestis* [40]. We found that transcription of *Ifnβ* was induced in the lungs early, and then declined. *Ifnar*-deficient mice were significantly more resistant to infection compared to wild type suggesting that IFN-I contributes to the pathogenesis of plague. Neutrophil depletion in the bone marrow became pronounced in wild type mice compared to *Ifnar^−/−^* even though both strains of mice initially developed a similar systemic infection. In contrast, the IFN-β transcription factor IRF-3 was required for host defense in a manner that was not dependent on IFN. Bacterial growth and inflammation proceeded more rapidly in *Irf3^−/−^* mice and *in vitro*, *Y. pestis* infection of bone marrow derived macrophages and neutrophils from the mutant mice resulted in decreased phagocytosis. Together, the data demonstrate the importance of IRF-3 to the basic process of phagocytosis, suggesting an interferon-independent role for the transcription factor during bacterial infection.

## Results

### Type I interferon is induced early following pulmonary *Y. pestis* infection

**Figure 1:**
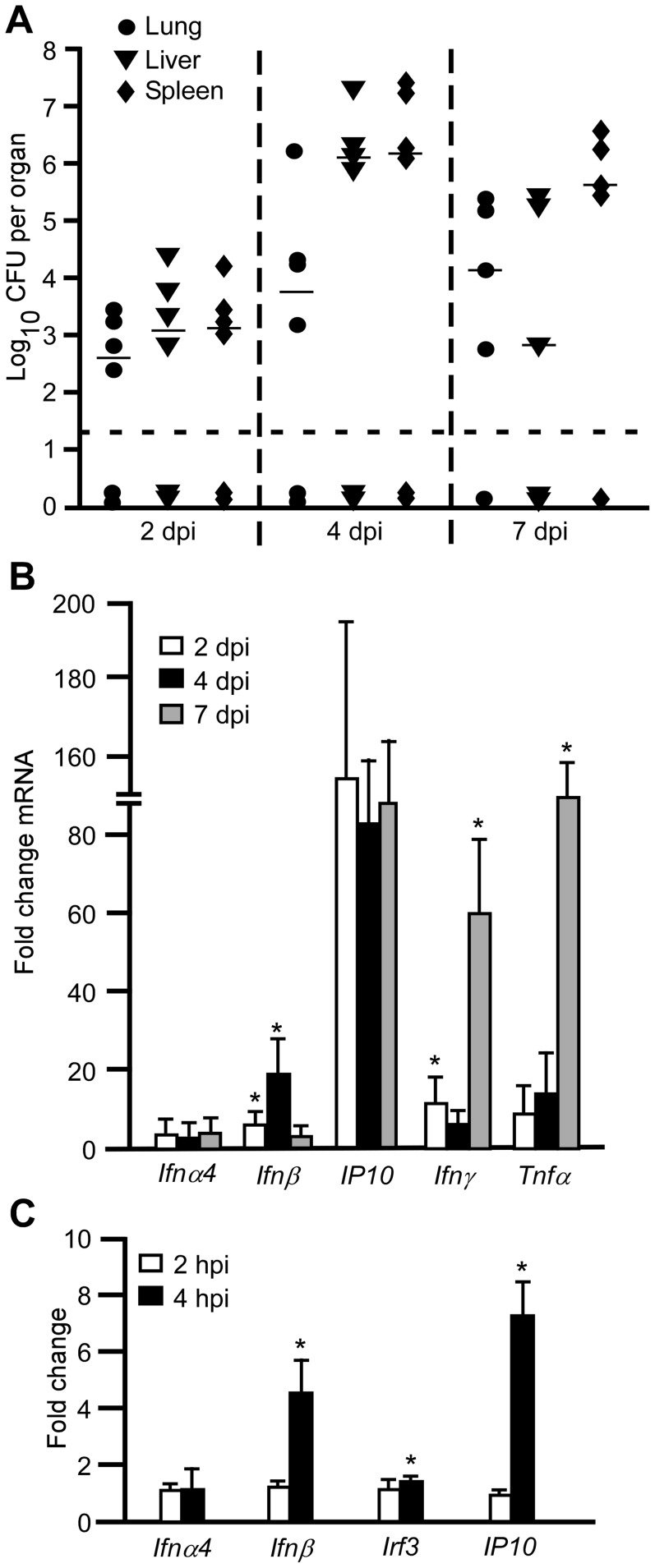
Type I interferon is produced following infection of C57BL/6 mice. (A–B) Wild type female C57BL/6 mice were challenged by intranasal infection with *Y. pestis* KIM D27. Groups of 3 mice were analyzed for bacterial load and gene expression on days 2, 4 and 7 post-infection. (A) Bacterial titer recovered in the lungs, liver and spleen. Bars depict the median titer (n = 6 total per group, 2 independent trials); *P<0.05 as determined by Kruskal Wallis rank sum test. (B) mRNA expression in the lungs. Gene expression values were normalized to the housekeeping gene *ywhaz* and are presented as a ratio compared to age-matched female C57BL/6 mice that were not infected. Data shown are a subset of a total of 17 analyzed from two independent experiments (n = 6 mice total per time point); error bars depict the standard deviation from the mean. (C) RAW 264.7 cells were infected by *Y. pestis* KIM D27, mRNA isolated and probed for expression of the indicated genes by real time PCR. Data were normalized to housekeeping gene *ywhaz*. Data shown is representative of two independent trials; *P<0.05 compared to not infected as determined by Wilcoxan matched pairs rank test.

Intranasal infection of non-pigmented (pgm^−^) strains of *Y. pestis* leads to lethal septicemic plague, with little to no bacterial growth in the lungs, effectively slowing the progression of disease [40]. In this model, mice that were pre-treated with inorganic iron more uniformly progressed towards lethal septicemic plague compared to untreated mice over 5–9 days. In order to identify host genes that may be important to *Y. pestis* infection, we challenged wild type C57BL/6 mice that had been pre-treated with iron with the pgm^−^ strain KIM D27 by intranasal infection. On days 2, 4 and 7, mice were euthanized, lungs, liver and spleen homogenized in sterile PBS and used to measure bacterial load and host gene expression. We found that bacteria disseminated early and could be recovered from the liver and spleen at 2 days post-infection after which they typically either replicated and caused lethal disease (5 of 6 mice survived until day 7) or appeared to be slowly clearing the infection (Figure 1A). As early as 2 days post-infection, we observed a significant increase in transcription of *Ifnβ* in the lungs which peaked on day 4 then declined (Figure 1B). *Ip10* (*Cxcl10*), a pro-inflammatory cytokine activated by IFN-β, also appeared induced in the lungs on day 2. Other IFN-responsive genes such as *Mx1* were also significantly increased on day 2 post-infection whereas *Ifnα* was not induced by *Y. pestis* (Table S1). In contrast, mRNA levels of cytokines *Ifnγ* and *Tnfα* peaked late during infection on day 7, a time where animals showed signs of acute disease. Together, it appears that *Yersinia pestis* activate type I IFN during pulmonary infection. Furthermore, similar to previous reports, we found the expression of type II IFN and the NF-κB-responsive *Tnfα* to be initially suppressed, only being activated after the onset of disease [28], [32].

RAW 264.7 cells, a monocyte-derived macrophage cell line, were infected with *Y. pestis* KIM D27 and expression of cytokines was measured by real time PCR. These results demonstrated a significant increase in expression of *Ifnβ* and *Ip10* mRNA at 4 hrs post-infection whereas *Ifnα4* was not induced (Figure 1C). Together the data suggest that macrophages produce IFN-β following infection by *Y. pestis* and may respond to it by activating expression of pro-inflammatory cytokines. We therefore sought to understand the role of IFN-β in the progression of plague.

### IFN-I signaling contributes to the pathogenesis of plague

**Figure 2:**
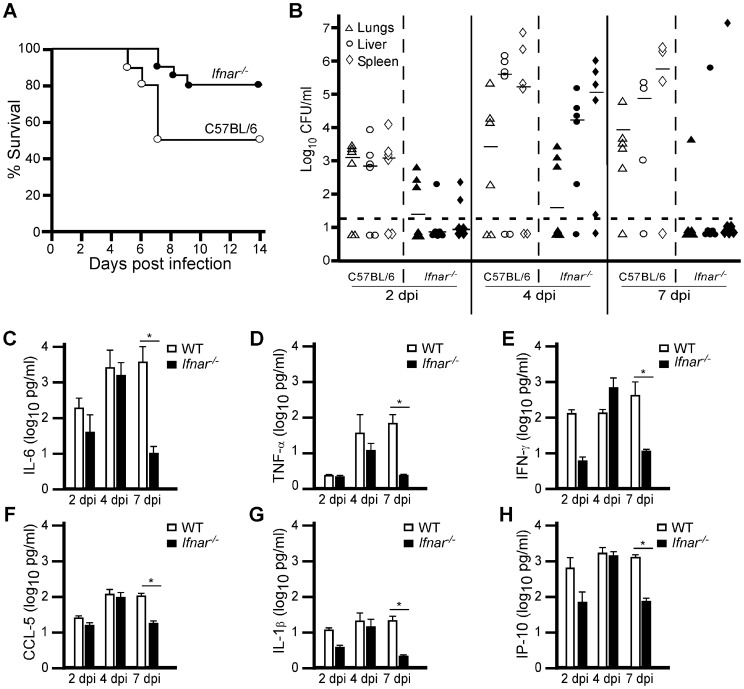
Type I IFN receptor deficient mice are more resistant to infection by *Y. pestis*. Groups of 5–10 wild type C57BL/6 and *Ifnar^−/−^* mice were challenged by intranasal infection of *Y. pestis* KIM D27. (A) Survival curve (collected from 3 independent trials, n = 30 for wild type mice, n = 20 for *Ifnar^−/−^* mice); P = 0.0378 compared to wild type mice, analyzed by Gehan Wilcoxan test (B) Bacterial load from lungs, liver and spleen on days 2, 4 and 7; P>0.05, analyzed by Kruskal Wallis rank sum test, for all tissues between strains (data shown are representative of two independent trials, n = 6 mice per group); (C–H) Serum from these mice was analyzed for 19 cytokines and chemokines; a subset of 6 are shown for a representative trial (two independent trials, n = 6 mice per group): (C) IL-6, (D) TNF-α, (E) IFN-γ, (F) CCL-5, (G) IL-1β, (H) IP-10. Open symbols represent wild type, closed symbols represent *Ifnar^−/−^* tissues; *P<0.1 wild type compared with *Ifnar^−/−^* mice; significance was determined by Kruskal Wallis rank sum test.

To understand the role of type I IFN signaling to *Yersinia pestis* infection, we studied susceptibility of mice lacking the IFN-I receptor, IFNAR, to pulmonary infection by *Y. pestis*. Mice lacking *Ifnar* were not more sensitive to infection, and in fact, they were more resistant with a significant reduction in mortality and increase in time to disease (Figure 2A). Bacterial growth in the lungs, liver and spleen of *Ifnar^−/−^* mice appeared similar to wild type early during infection and in each, growth by more than 3 orders of magnitude was seen between days 2 and 4 (Figure 2B). In contrast, wild type mice continued to progress and increased bacterial titers were recovered on day 7 whereas bacterial clearance in *Ifnar^−/−^* mice had occurred at this time point and only one mouse had recoverable bacteria.

We also examined serum cytokines in these mice. Along with bacterial titer, pro-inflammatory cytokines in the serum of both strains of mice were similar early and no gross differences were apparent on days 2 and 4 post-infection for any cytokine (Figure 2C–H). By day 7, most pro-inflammatory cytokines were lower in *Ifnar^−/−^* mice compared to wild type, consistent with their reduction in bacterial load. Together these data suggest that IFN-I signaling becomes detrimental during the later stage of infection.

**Figure 3:**
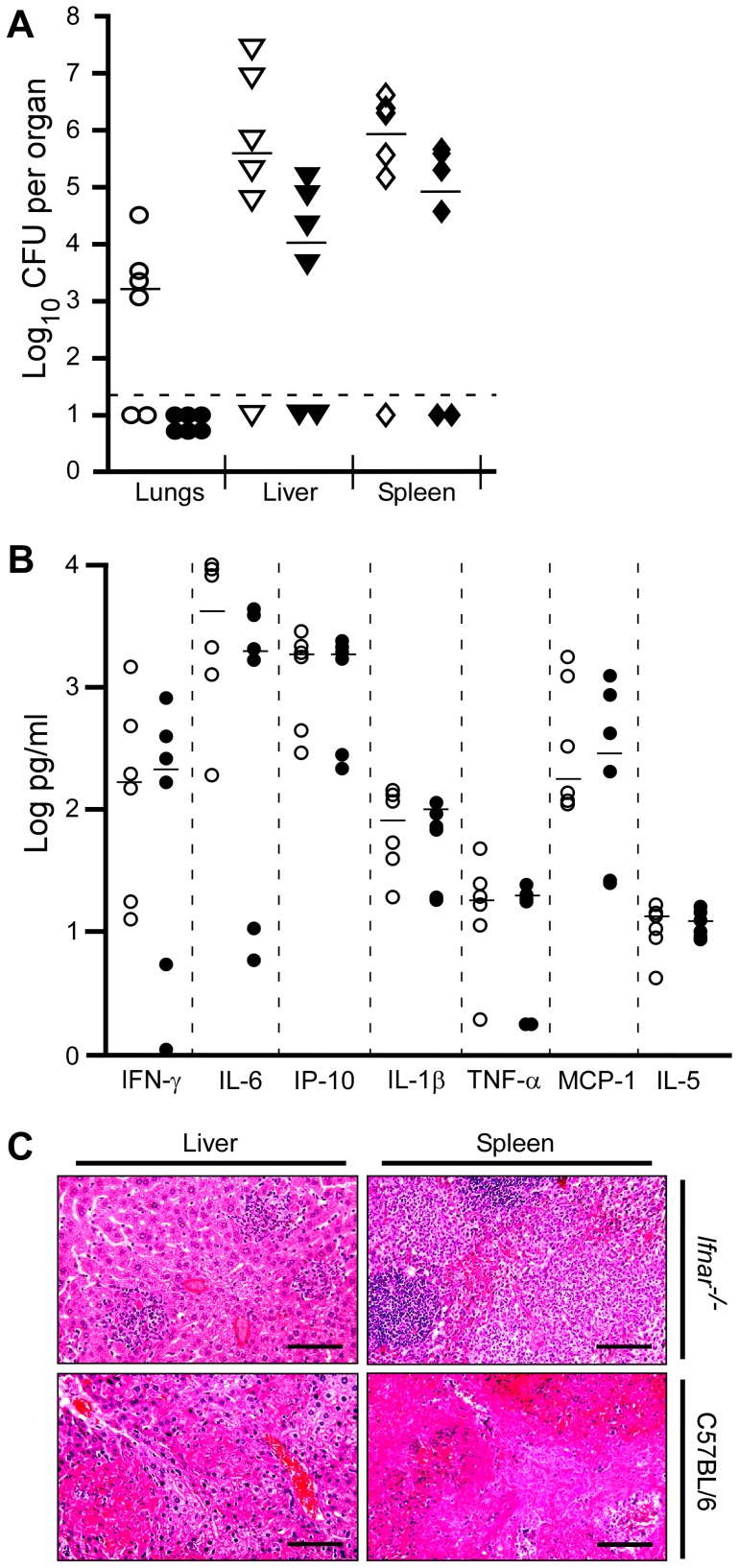
IFNAR dependent depletion of neutrophils correlates with disease. Wild type C57BL/6 and *Ifnar^−/−^* mice were challenged by intranasal infection of *Y. pestis* KIM D27. On day 5 post-infection, 6 mice per group were euthanized and tissues processed for (A) bacterial load, (B) serum cytokines and (C) histology. Samples for all assays were obtained for each mouse; open shapes are wild type, closed shaped are *Ifnar^−/−^* mice; representative data are shown; n = 12 mice per strain assayed in 2 independent trials. All values for titers and cytokines were not statistically significant (P>0.9) between wild type and *Ifnar^−/−^* mice as determined by Kruskal Wallis rank sum test. (C) H&E stains of *Ifnar^−/−^* liver and spleen (top panels, left to right) and wild type liver and spleen (bottom panels, left to right). Images shown are representative of the mice with positive bacterial titers. Scale bar indicates 100 µm.

Given that differences in host responses and bacterial clearance were evident between days 4 and 7 post-infection, we assayed the infection on day 5 in more detail to identify the host responses that differed between strains and determine how these influenced disease pathology. Similar to what we observed on days 2 and 4, bacterial load in the lung, liver and spleen on day 5 appeared reduced in *Ifnar^−/−^* mice compared to wild type but these differences were not statistically significant (Figure 3A). In addition, serum samples revealed similarities in the secretion of pro-inflammatory cytokines (Figure 3B shows a subset of cytokines measured). Fixed tissues from these mice were examined by histopathology and immunohistochemistry. Pathological analysis indicated similarities in overall severity of lesions and degree of inflammation between wild type and mutant, with correlation between increased bacterial titers and disease progression (Figure 3C). Upon further examination, we found a correlation between infiltration of polymorphonuclear cells with reduced bacterial titer in the liver and spleen of *Ifnar^−/−^* mice whereas for wild type mice, polymorphonuclear cells appeared less common in these tissues. Immunohistochemistry suggested these PMNs were Gr-1^+^ and therefore were likely neutrophils and/or monocytes (Figure S1). Caspase-3 staining appeared similar in both the liver and spleen of wild type and *Ifnar^−−/−^* mice, with the most pronounced staining in the necrotic lesions (data not shown). Together, the results suggest a systemic, IFN-β-dependent reduction in neutrophil/monocyte populations in wild type mice in the late stage of infection.

**Figure 4:**
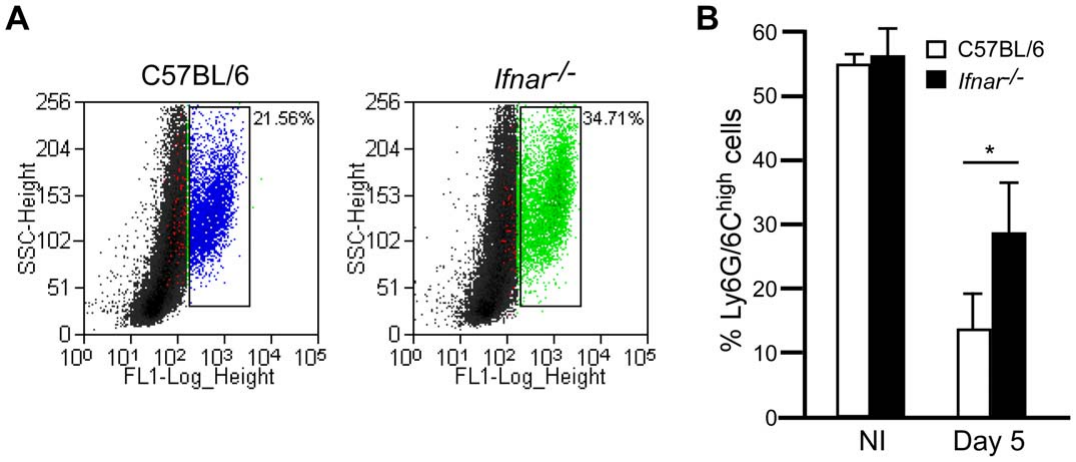
IFNAR-dependent depletion of Ly6G^+^ cells in the bone marrow. Bone marrow cells of wild type C57BL/6 and *Ifnar^−/−^* mice that were not infected (NI) (n = 3 mice per group assayed in 2 independent trials) or on day 5 post-challenge by intranasal infection with *Y. pestis* KIM D27 (n = 13 mice per group assayed in 3 independent trials) and stained with anti-Ly6G/6C-FITC followed by flow cytometry analysis. Data was gated on high stained cells as indicated in the scatter plot (A) and are shown for mice with the greatest percentage neutrophils in each group from a single trial. (B) Mean percent neutrophils from representative trial, error bars indicate the standard deviation from the mean. White bars indicate wild type mice, black bars indicate *Ifnar^−/−^* mice; *P<0.05 between wild type and *Ifnar^−/−^* mice determined by two-tailed Kruskal Wallis test.

Since we observed an apparent systemic neutropenia that might be dependent on IFNAR, we asked whether neutrophils in the bone marrow were also reduced in wild type mice as disease progressed. Bone marrow was isolated from tibia and femurs, then fixed and stained with anti-Ly6G/6C and the cells examined by flow cytometry. Bone marrow from wild type and *Ifnar^−/−^* mice that were not infected contained similar numbers of neutrophils (Ly6G^hi^, Figure 4). However, on day 5 post-infection, wild type mice harbored significantly fewer Ly6G^hi^ cells in the bone marrow compared to *Ifnar^−/−^* mice. Thus, on day 5, neutropenia is more pronounced in wild type mice than in *Ifnar^−/−^* mice. This observation may explain why wild type mice lose control over the infection, while *Ifnar^−/−^* mice are able to contain it. Together these data support a model whereby IFNAR signaling contributes to the depletion of immune cells during *Y. pestis* infection.

### *Irf3^−/−^* mice develop septicemic plague at an accelerated rate

**Figure 5:**
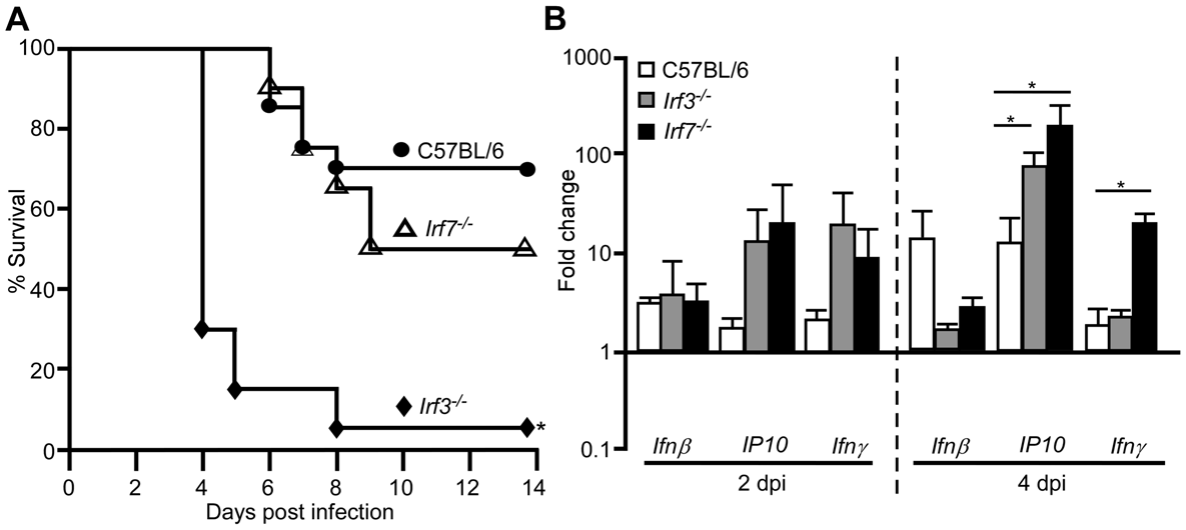
*Irf3^−/−^* mice are more sensitive to pulmonary infection by *Y. pestis*. (A) Groups of 5–7 male and female wild type C57BL/6, *Irf3^−/−^*, and *Irf7^−/−^* mice were challenged by intranasal infection of *Y. pestis* KIM D27 and monitored for 14 days; *P<0.05 compared to wild type as determined by Gehan-Wilcoxan test; data shown are combined from three independent experiments for each strain (n = 20 for wild type, n = 13 for *Irf3^−/−^* mice, n = 11 for *Irf7^−/−^* mice). (B) Groups of 3 wild type C57BL/6, *Irf3^−/−^* and *Irf7^−/−^* mice were challenged by intranasal infection with *Y. pestis* KIM D27. On days 2 and 4 post-infection, mice were euthanized by CO_2_ asphyxiation, lungs harvested and processed for RNA isolation and real time PCR. mRNA expression values were normalized to housekeeping gene *ywhaz* [19]; y-axis represents fold change compared to mice that were not infected. Error bars depict the standard deviation from the mean. Data shown were collected in a single trial (n = 3 for all day 2 samples; only 2 *Irf3^−/−^* and *Irf7^−/−^* mice survived until day 4, n = 3 for wild type C57BL/6 on day 4). *P<0.05, ***P<0.005 as determined by one way ANOVA followed by Bonferroni's multiple comparison test.

We also studied the sensitivity of mice lacking the IFN-β transcription factors *Irf3* or *Irf7*. We challenged wild type, *Irf3^−/−^* and *Irf7^−/−^* mice by intranasal infection of *Y. pestis* KIM D27 and followed development of acute disease over a 14 day period. At this challenge dose, 30% of wild type mice developed lethal disease on days 5–9 post-infection (Figure 5A). Similarly, 50% of *Irf7^−/−^* mice developed plague with a similar time to lethal disease. However, *Irf3^−/−^* mice were significantly more sensitive and 90% developed lethal disease in only 4 days. Pre-treatment of mice with iron did not substantially impact development of disease in *Irf3^−/−^* mice as those that did not receive iron also developed an increased rate of mortality compared to wild type (Figure S2).

To determine whether IRF-3 was responsible for synthesis of type I IFN during *Y. pestis* infection, we challenged wild type, *Irf3^−/−^* and *Irf7^−/−^* mice with *Y. pestis* KIM D27 and measured gene expression in the lungs on days 2 and 4 post-infection. While it appeared that *Ifnβ* expression was decreased in *Irf3^−/−^* and *Irf7^−/−^* mice compared to wild type on day 4 post-infection, expression of this cytokine was not fully dependent on either transcription factor (Figure 5B). Similarly, *Ip10* expression was not dependent on IRF-3 or IRF-7, and in fact, increased expression of *Ip10* was observed in both strains of mutant mice. Thus, *Ifnβ* expression is not likely to depend on IRF-3 or IRF-7 following *Y. pestis* infection of the lung.

### IRF-3 contributes to the control of bacterial growth *in vivo*

**Figure 6:**
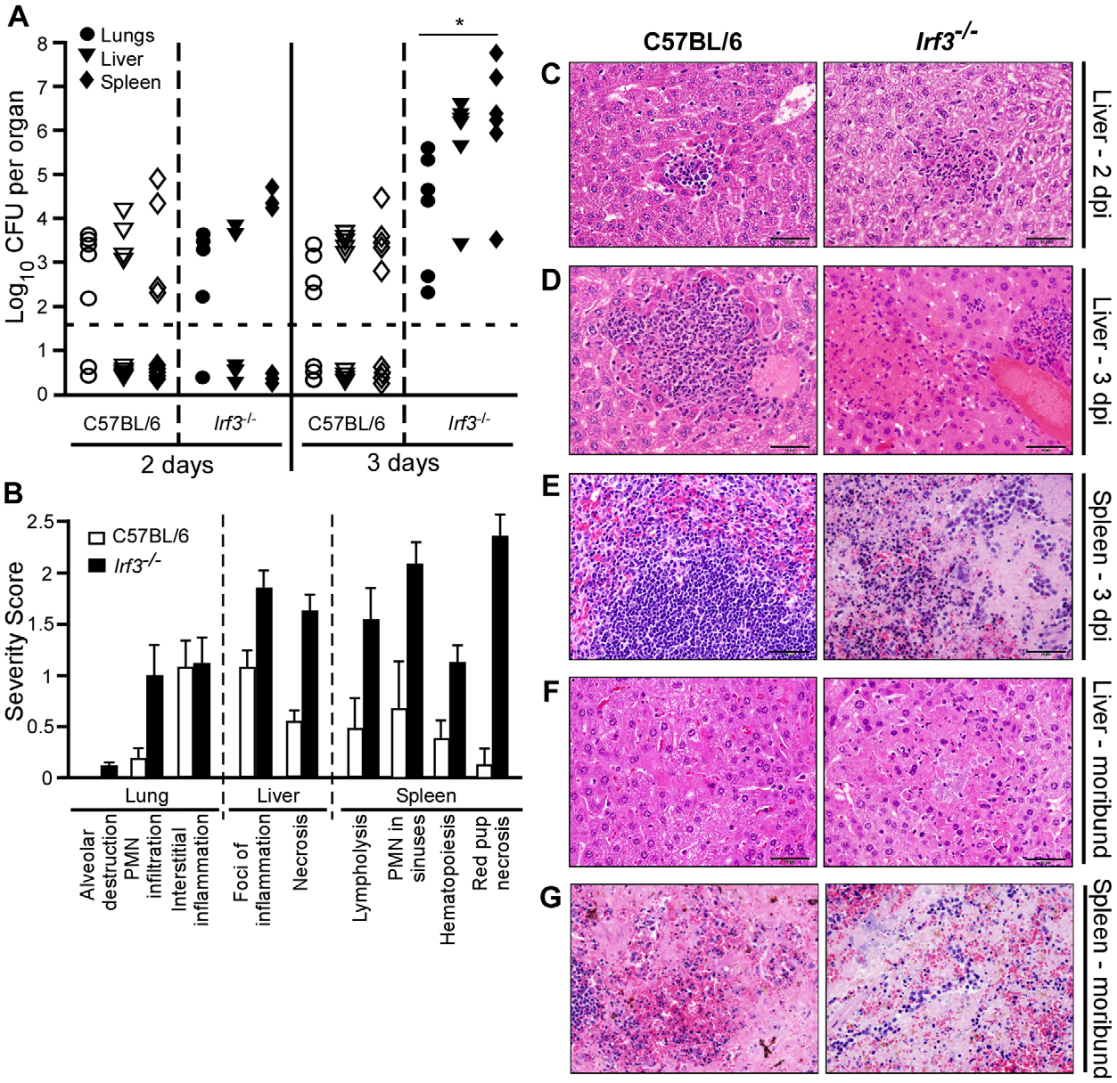
*Irf3^−/−^* mice develop septicemic plague at an accelerated rate. Groups of 3 wild type C57BL/6 and *Irf3^−/−^* mice were challenged by intranasal infection of *Y. pestis* KIM D27. (A) Bacterial load for lungs, liver and spleen of wild type and *Irf3^−/−^* mice on days 2 and 3 post-infection; open symbols indicate wild type, closed symbols indicate *Irf3^−/−^*; *P<0.05 for all three tissues compared to wild type as determined by Kruskal Wallis rank sum test. (B) Mean severity scores for histopathology of lungs, liver and spleen on day 3; error bars indicate the standard deviation from the mean scores. (C–G) Wild type tissues are the left panels, *Irf3^−/−^* tissues are the right panels; Day 2 (C), 3 (D–E), and moribund (F–G) hematoxylin and eosin (H&E) stain for liver (C–D,F) or spleen (E, G). Scale bar indicates 50 µm. Data shown are representative from 2 independent experiments (n = 6 mice per strain).

Bacterial load was examined in the lungs, liver, and spleen of wild type and mutant mice on days 2, 3 and 4 post-infection. Results showed similar bacterial titers in all three tissues on day 2 post-infection, and in several animals in all groups the bacteria were undetectable (Figure 6A, Figure S3). In striking contrast, rapid bacterial growth occurred over the next 24 hrs in *Irf3^−/−^* mice but not in wild type or *Irf7^−/−^* mice.

We also examined disease pathology by staining formalin fixed lungs, liver and spleen with hematoxylin and eosin (H&E). Total pathological severity scoring of lungs, liver and spleen indicated a disease in *Irf3^−/−^* mice consistent with bacterial sepsis on day 3 post-infection, with a large degree of necrosis in infected tissues and increased inflammation, while wild type mice appeared to have less necrosis and inflammation (Figure 6B). All mice harbored mild lesions on day 2 post-infection, and in the liver of *Irf3^−/−^* mice there were increased inflammatory foci, many of which contained dying cells while in wild type mice small neutrophilic foci formed that typically contained intact cells (Figure 6C). Disease in *Irf3^−/−^* mice progressed rapidly and on day 3, degenerating neutrophilic inflammatory foci were still present in the liver, but multiple large necrotic lesions were also observed in both the liver and spleen (Figure 6D–E). In contrast, neutrophilic foci in the liver of wild type mice remained comprised primarily of intact cells and there was minimal damage to the spleen. Bacterial colonies could be seen in some areas of necrosis in the liver of mutant mice and these appeared larger and more numerous as disease progressed suggesting that rapid bacterial growth caused accelerated plague in the *Irf3^−/−^* mice (Figure 6F). Noticeably, intact neutrophils were generally absent in areas of bacterial colonies which were instead surrounded by necrotic tissue. Likewise, spleens were necrotic in moribund mice (Figure 6G), and, overall, pathology in moribund mice appeared similar in both groups. Lungs of wild type mice had neither inflammation nor disease while *Irf3^−/−^* mice had developed mild inflammation in the lungs on day 3 (Figure S4). Together the data suggest that *Irf3^−/−^* and wild type mice succumbed to septicemic plague though the mutant mice developed severe disease more rapidly. In contrast to *Irf3^−/−^* mice, all tissues of *Irf7^−/−^* mice examined had fewer lesions, mild to moderate inflammatory foci, and were indistinguishable from wild type (Figure S5).

**Figure 7:**
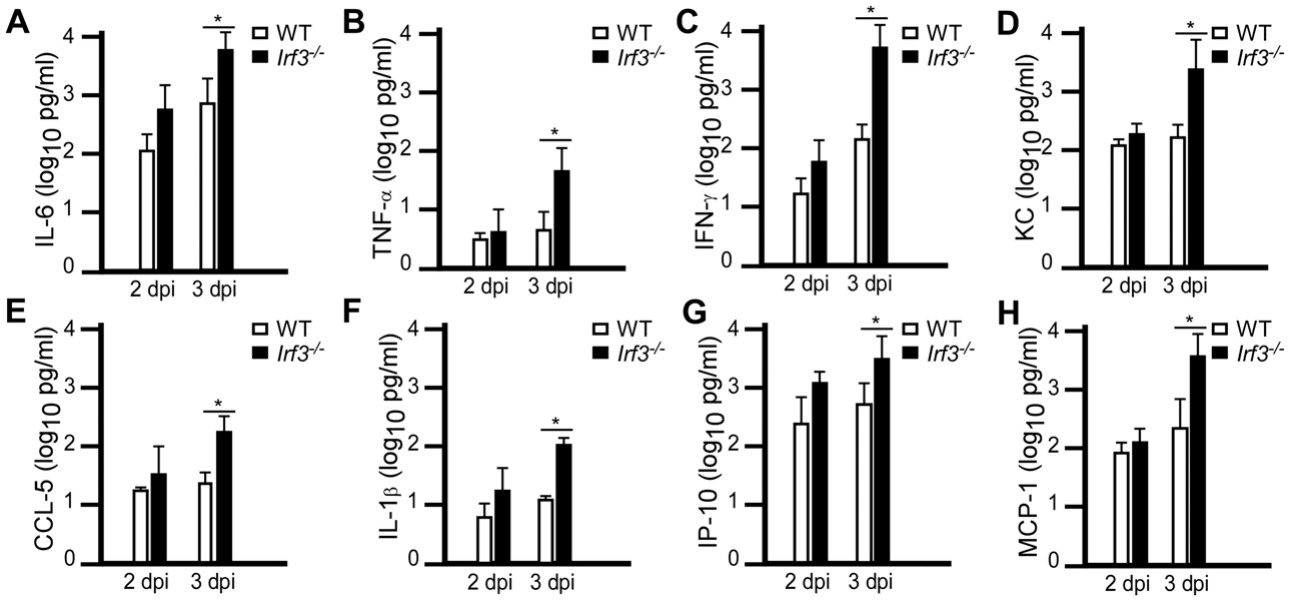
*Irf3^−/−^* mice express pro-inflammatory responses that correlate with disease progression. Wild type C57BL/6 and *Irf3^−/−^* mice were challenged by intranasal infection of *Y. pestis* KIM D27. On days 2 and 3 post-infection, serum was collected by cardiac puncture and analyzed by cytokine bead array. A subset of 8 of the 19 cytokines measured are shown, open bars are from wild type mice; closed bars are from *Irf3^−/−^* mice: (A) IL-6, (B) TNF-α, (c) IFN-γ, (D) KC, (E) CCL-5, (F) IL-1β, (G) IP-10, (H) MCP-1. *P<0.05 as determined by Kruskal Wallis rank sum test; data shown are representative from two independent experiments, concurrent with the samples shown in figure 3 (n = 6 mice per strain).

We also analyzed serum cytokine production in wild type and *Irf3^−/−^* mice on days 2 and 3 post-infection. Both strains harbored relatively low levels of pro-inflammatory cytokines in the serum on day 2 with no statistical significance between wild type and *Irf3^−/−^* mice for all cytokines analyzed (Figure 7A–H). In striking contrast, however, *Irf3^−/−^* mice produced significantly higher levels of pro-inflammatory cytokines in the serum on day 3 post-infection compared to wild type, whereas IL-17, IL-5 and MIP-1α were not detectably different between the strains (data not shown). Together, the data suggest a burst in the production of inflammatory cytokines occurred between days 2 and 3 post-infection for *Irf3^−/−^* mice. Since this is the same time point where both bacterial growth and tissue necrosis became substantially greater in *Irf3^−/−^* mice compared to wild type, these data suggest that bacterial growth or the subsequent tissue injury may have triggered a massive pro-inflammatory response.

Immunohistochemistry confirmed the identity of macrophages, monocytes and neutrophils and revealed the presence of apoptosis in the red pulp of the spleen (Figure S6). Inflammatory foci of the liver appeared to contain both macrophages and neutrophils and there was no caspase-3 staining in the degenerated foci. In the spleen, red pulp necrosis that stained positive for cleaved caspase-3 appeared to involve both macrophages and neutrophils. Together the phenotypic data suggest that *Irf3^−/−^* mice experience more extensive and accelerated tissue damage that correlates with an increase in bacterial growth.

### IRF-3 limits intracellular bacterial growth in bone marrow derived macrophages

**Figure 8:**
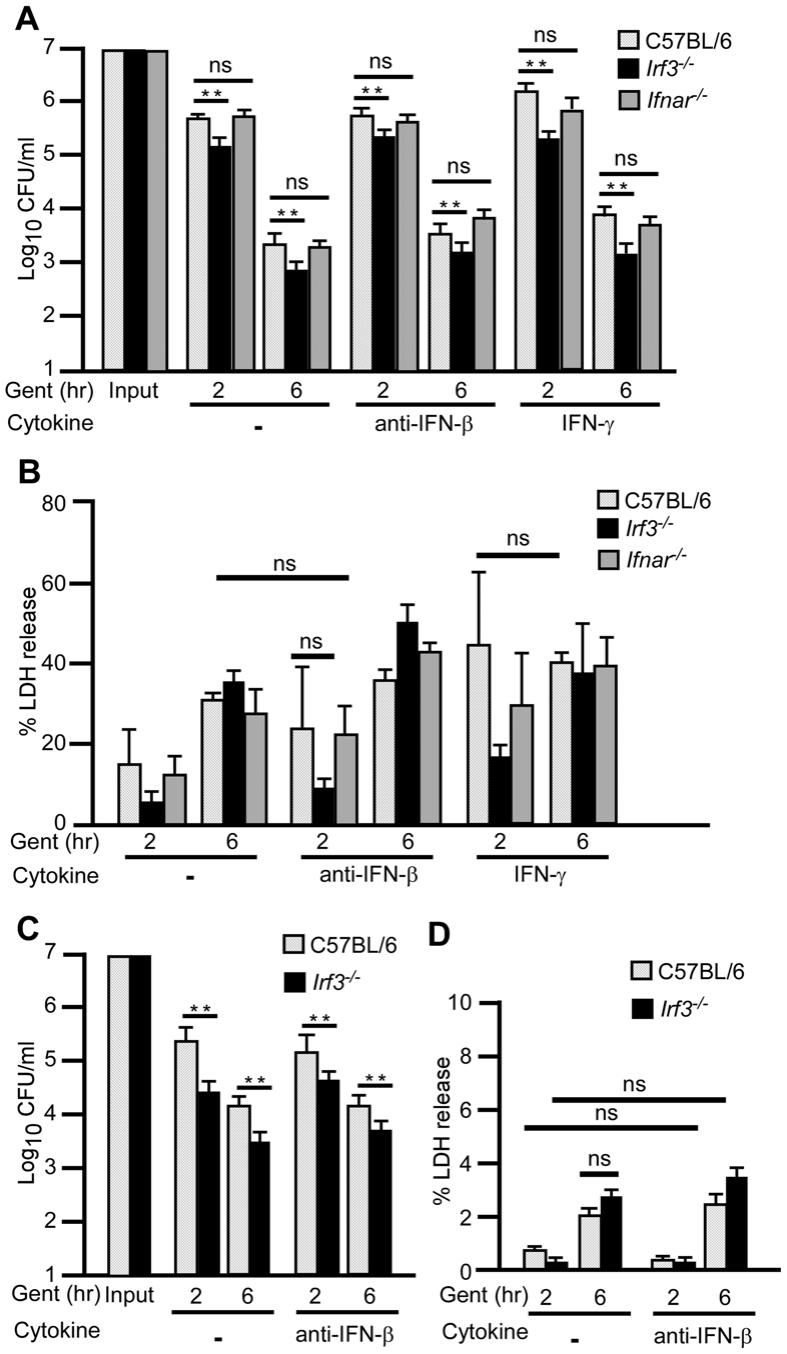
Decreased phagocytosis of *Y. pestis* in bone marrow derived macrophages (BMMs) and neutrophils (BMNs) from *Irf3^−/−^* mice. BMMs (A–B) or BMNs (C–D) from wild type and mutant mice were pre-treated with PBS, anti-IFN-β or IFN-γ before infecting with 1×10^7^ CFU *Y. pestis* KIM D27 at MOI of 10. At 30 min post-infection, gentamicin was added to the medium and the infection continued for a total of 6 hrs. Bacteria were enumerated by plating in triplicate (A, C) following detergent lysis of macrophages at t = 2 and 6 hr post-infection. Cell death was also assessed by collecting the culture supernatant at the same time points prior to detergent lysis and measuring the percent of LDH released compared to lysed control cells that were not infected (B, D). Data shown indicate the mean values collected in 2 independent trials; each sample was run in triplicate in each trial; error bars indicate the standard deviation from the mean. **P<0.005, significance was determined by one way ANOVA followed by Bonferroni's multiple comparison test (BMMs) or two-tailed Wilcoxan rank sum test (BMNs).

Previous work has shown that IFN-I helps limit intracellular growth of *L. pneumophila* in alveolar epithelial cells while, during *L. monocytogenes* infection of macrophages, IFN-I signaling reduces expression of IFN-γ receptor thereby preventing activation of macrophages by IFN-γ [13], [14]. We therefore sought to determine whether IRF-3 and IFN-I had an impact on bacterial uptake and survival in macrophages. Towards this end, we isolated bone marrow derived macrophages (BMDMs) from wild type, *Irf3^−/−^* and *Ifnar^−/−^* mice and performed a gentamicin protection assay to enumerate intracellular bacteria following infection. BMDMs were pre-treated with PBS, anti-IFN-β or IFN-γ for 4 hrs prior to infection with *Y. pestis* KIM D27. Bacteria (1×10^7^ CFU) grown at 37°C were added to the macrophages at a multiplicity of infection of 10 (time 0) and incubated for 30 min before adding gentamicin to kill extracellular bacteria. After 90 min incubation in gentamicin (time 2 hr), macrophages were lysed and intracellular bacteria enumerated by plating on agar medium. The results showed that 5–6% of *Y. pestis* was intracellular in wild type and *Ifnar^−/−^* BMDMs while only 2–3% of *Y. pestis* was intracellular in *Irf3^−/−^* BMDMs, a difference that was reproducible and statistically significant (Figure 8A). This happened in the presence and absence of anti-IFN-β showing that IFN-β signaling does not appear to affect phagocytosis. Further, BMDMs from *Ifnar^−/−^* mice had no defect in phagocytosis compared to wild type. Treatment with IFN-γ yielded results similar to untreated in all mouse strains. BMDMs from wild type and mutant mice were able to kill intracellular *Y. pestis* similarly as approximately 0.5–1% of the bacteria found at 2 hrs were still present at 6 hrs post-infection. IFN-γ treatment had little effect in all three mouse strains, with each showing 0.5–1% recovery of bacterial titer between 2 and 6 hrs post-infection. These data suggest *Irf3^−/−^* macrophages have a defect in an early stage of phagocytosis. We also measured cell death in these samples by determining LDH release caused by the infection compared with detergent-lysis of BMDMs. These results showed no detectable differences in cytotoxicity between wild type, *Ifnar^−/−^* and *Irf3^−/−^* mice suggesting that macrophage viability following infection is not dependent on IRF-3 (Figure 8B).

Neutrophils play a key role in restriction of *Y. pestis* growth *in vivo* [41]. Furthermore, neutrophilic function is modified following injection of YopH by the *Yersinia* type III secretion system, a virulence mechanism that blocks intracellular calcium signaling and contributes to resistance of extracellular bacteria to neutrophilic killing [42]. We therefore wondered if the increase in susceptibility of *Irf3^−/−^* mice to plague might be due to a defect in bacterial phagocytosis by neutrophils. To address this, we measured phagocytosis of *Y. pestis* by bone marrow derived neutrophils (BMNs) from wild type and *Irf3^−/−^* mice. Similar to macrophages, approximately 10% of the bacteria were taken up by neutrophils from wild type mice whereas only 1% of the infecting dose was taken up by *Irf3^−/−^* neutrophils suggesting IRF-3 is necessary for phagocytosis (Figure 8C). However, once internalized, neutrophils from *Irf3^−/−^* bone marrow were capable of killing *Y. pestis* to a similar degree as wild type neutrophils. When examined for cell death by LDH release, BMNs showed no detectable differences between wild type or *Irf3^−/−^* cells during this time period (Figure 8D). Further, no significant differences were detected in anti-IFN-β treated compared to untreated BMNs from either wild type or *Irf3^−/−^* mice over the 6 hr time period examined. Together these data suggest that IRF-3 may be required for efficient phagocytosis of *Y. pestis*. Further, since anti-IFN-β did not affect phagocytosis, it appears the effect of IRF-3 on this pathway is not mediated by IFN-β. Since neutrophils are known to be important to host defense against *Y. pestis*, these data suggest that a defect in phagocytosis may be responsible for the increase in susceptibility of *Irf3^−/−^* mice [43].

### IRF-3-mediated protection is lost in the presence of the *Y. pestis* pigmentation locus

**Figure 9:**
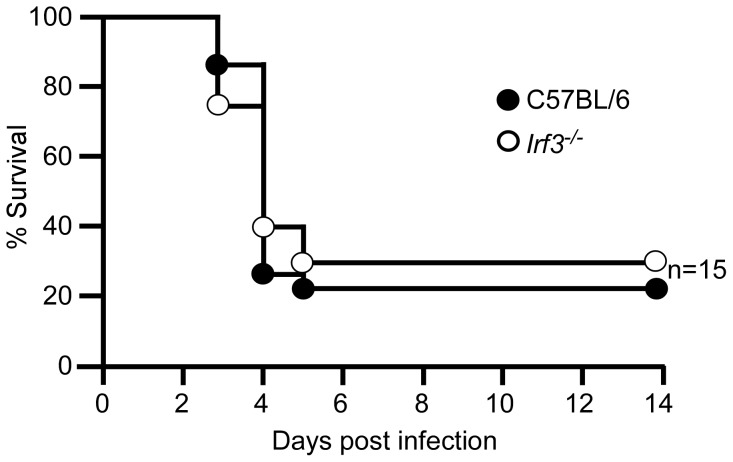
Increase in sensitivity of *Irf3^−/−^* mice is dependent on the absence of the *Y. pestis* pigmentation locus. Groups of five male and female wild type C57BL/6 and *Irf3^−/−^* mice were challenged by intranasal infection of 1×10^4^ CFU *Y. pestis* KIM5^+^ and monitored over 14 days for development of disease. Data shown were collected in 3 independent experiments, n = 5 mice per group in each experiment; 15 mice total per strain. P = 0.7295 between wild type and *Irf3^−/−^* mice as determined by Mantel Cox log rank test.

Our septicemic plague model involves intranasal delivery of the non-pigmented laboratory strain *Y. pestis* KIM D27 which lacks 102 kb of chromosomal DNA including a high pathogenicity island [44]. We therefore wanted to understand the relevance of IRF-3 following challenge with fully virulent *Y. pestis*. Intranasal infection of the wild type *Y. pestis* strain KIM5 leads to primary pneumonic plague over a period of 3–4 days, and *Irf3^−/−^* mice succumbed to disease with an indistinguishable time course and mortality rate (Figure 9). Similar results were obtained following challenge with *Y. pestis* CO92, another fully virulent strain from the *Orientalis*, rather than *Mediavalis*, biovar (data not shown). Together the data suggest that the wild type bacteria bypass IRF-3 either because its contribution to host defense is less pronounced against the fully virulent bacteria or wild type *Y. pestis* carry virulence factors in the pgm locus that silence IRF-3's role in host defense.

We also tested an isogenic non-pigmented mutant strain of KIM5 isolated by plating on congo red agar to verify that the role of IRF-3 is not limited to a lab-adapted *Y. pestis* strain isolated in 1965 [45]. This strain (KIM5^−^) was then used to challenge wild type and *Irf3^−/−^* mice by intranasal inoculation. Similar to D27, challenge with *Y. pestis* KIM5^−^ led to an apparent acceleration of disease in *Irf3^−/−^* mice compared to wild type, though for the single trial we performed, these differences were not significant (p = 0.28, Figure S7). These data suggest that the role of IRF-3 in host defense is not specific to bacterial strain isolate and support a role for IRF-3 in preventing septicemic plague.

## Discussion

*Yersinia pestis* requires high blood titer to enable its transmission to fleas and persistence in the environment [46]. Its virulence, therefore, has evolved to promote replication to high titers in the blood, an environment that becomes overwhelmed by anti-bacterial defense mechanisms that attempt to prevent this massive growth. Multiple interactions between virtually all arms of the innate immune system and *Yersinia* determine the outcome of infection, with each having downstream consequences that may contribute further to disease. In this work, we addressed the role of type I interferon (IFN-I) activation in mice following *Y. pestis* infection. We identified production of IFN-β in the lungs early following pulmonary infection and found this was not dependent on the transcription factors IRF-3 or IRF-7. Production of type I IFN led to increased susceptibility to plague in a manner that correlated with systemic neutrophil depletion. IRF-3, however, was necessary for host defense and its expression led to decreased susceptibility to plague in a manner that correlated with decreased bacterial growth. Together these data raise new insight into the innate immune response.

**Figure 10:**
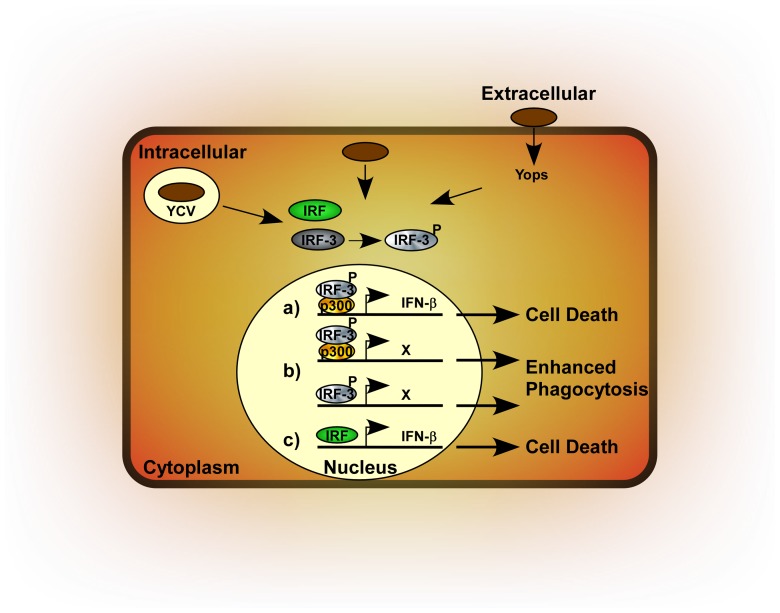
Model for *Yersinia pestis* activation of IRF-3 and type I interferon. Extracellular bacteria inject Yops via the type III secretion system into the host cell where they may activate signaling pathways that terminate in IRF-3 phosphorylation. Bacteria may also be taken up by phagocytosis where they are able to survive in *Yersinia* containing vacuoles or within the cytoplasm where they may be detected by pattern recognition receptors leading to IRF-3 phosphorylation. Following phosphorylation, IRF-3P migrates to the nucleus where it may bind p300 (a) or may form an alternative DNA binding complex (b) and activate transcription of IFN-β and other, currently unknown genes (X). IFN-β expression may also be dependent on other interferon response factors (IRFs) (c) and IFN-β signaling leads to neutropenia perhaps by activation of programmed cell death. Phagocytosis is enhanced by the product of gene X.

Wild type *Y. pestis* can survive and even replicate in macrophages yet cause disease due to exponential growth of extracellular bacteria that secrete cytotoxins (Yops) and other virulence factors into host cells [34], [47], [48], [49]. Thus, the bacteria interface with the host in multiple environments where they may be recognized by surface-located and intracellular pattern recognition receptors or may perturb intracellular signaling through direct interactions involving the Yops of the type III secretion system (Figure 10). Bacterial pathogen recognition by one or more of these pathways, leads to phosphorylation of IRF-3 and perhaps also activation other interferon regulatory factors. Phosphorylation of IRF-3 causes its migration to the nucleus where it can form an active DNA binding complex, likely with p300, but possible alternative DNA binding complexes cannot be ruled out. In this manner, IRF-3 not only activates transcription of IFN-β but may also stimulate up-regulation of key mediators of phagocytosis. Additional work is needed to identify genes that are affected by IRF-3, whether the same IRF-3 has alternative phosphorylation or interactions in the nucleus that affect its activity or if other interferon regulatory factors are responsible for IFN-β production.

IRF-3 phosphorylation allows its nuclear migration and multiple phosphorylation sites of IRF-3 have been demonstrated [6]. TRIF activation by other bacterial pathogens typically leads to the production of IFN-β which can limit the infection by stimulating the expression of pro-inflammatory cytokines and chemokines such as IP-10 or RANTES, thereby inducing neutrophil recruitment [20], [50]. Here we found evidence that phagocytosis may require IRF-3 activation. Though we do not yet know if this requirement is caused by production of a secreted factor such as a cytokine or other inflammatory mediator, expression of proteins involved in bacterial uptake, or even if additional neutrophil or macrophage functions are dependent on IRF-3, it is clear that IRF-3 may have a greater number of target genes than previously appreciated. Phosphorylated IRF-3 is a potent transcription factor with affinity for the conserved interferon stimulated response element (ISRE) on the promoters of hundreds of target genes in addition to IFN-β. IFN-independent anti-viral gene expression has been previously described involving IRF-3 and many of these induced proteins have been identified including those that regulate translation initiation, cell proliferation, apoptosis of infected cells, and others with unknown function [51], [52], [53], [54]. Regulation of these genes is complex, with different cell types and different tissues activating distinct genes through IRF-3 and it is clear that the method of stimulation of IRF-3 following viral infection can lead to different programs of downstream gene expression [55]. Future experiments addressing IRF-3-dependent, IFN-independent gene expression during *Y. pestis* infection will be important to identify the proteins that are necessary for the antibacterial effects of IRF-3.

Wild type *Y. pestis* are known to survive better than pgm^−^ strains inside activated macrophages and, in our analyses, we showed that the absence of IRF-3 had minimal effect on protection against infection by wild type bacteria. It is conceivable that the wild type pulmonary infection, which results in death due to acute bronchopneumonia, progresses so rapidly that IRF-3 protection is overwhelmed by the developing lung injury or that resident phagocytic cells of the lung are unable to activate the anti-bacterial defense mechanism mediated by IRF-3. Alternatively, IRF-3-dependent phagocytosis may be neutralized by one or more gene products encoded by the pgm locus. Pgm-encoded *ripA* is a virulence factor necessary for intracellular bacterial growth, but not phagocytosis, in IFN-γ activated bone marrow derived macrophages [56]. At least two additional virulence factors are believed to be encoded in the pgm locus, only one of which is known: the siderophore, yersiniabactin, whose deletion severely attenuates virulence [57]. Future studies combining mouse and bacterial genetics will facilitate an understanding of the mechanism whereby wild type *Y. pestis* induce or evade IRF-3.

Our results suggest that it is likely that multiple transcription factors activate IFN-I during *Y. pestis* infection, as the absence of IRF-3 or IRF-7 did not result in a complete loss of *Ifnβ* production. Recently, expression of IRF-1 was shown to be induced following *Y. pseudotuberculosis* infection though the role of this transcription factor during *Yersinia* infection has not been investigated [39]. Thus, it is conceivable that IRF-1 or even another IRF is responsible for IFN-β production. Alternatively, it may be that small amounts of IFN-I that remain in *Irf3^−/−^* and *Irf7^−/−^* mice are sufficient to cause immune cell depletion such that deletion of multiple IRFs are required to achieve the phenotype caused by deletion of IFNAR.

During *Listeria* infection, surface expression of IFN-γ receptor is down-regulated on macrophages making them less able to kill intracellular bacteria. We therefore analyzed phagocytosis and bacterial killing in macrophages lacking IFN-I signaling in the presence and absence of IFN-γ activation. In these experiments, we did not detect differences between wild type and *Ifnar* mutant macrophages in their ability to be activated by IFN-γ. Further, we did not observe a decrease in the production of pro-inflammatory cytokines such as KC or MCP-1 that are normally IFN-γ-dependent in the *Ifnar^−/−^* mice. Together these data suggest that the IFN-γ receptor is not down-regulated by IFN-I during *Y. pestis* infection. It is therefore likely that the downstream effects of IFN-I are somewhat pathogen-specific.

Our data found a link between IFN-I and neutrophil depletion and we propose that either neutrophils or another population responsible for their maturation and/or recruitment undergoes cell death in response to IFN-I signaling during *Y. pestis* infection (Figure 10). Depletion of neutrophils or other immune cells that is associated with type I IFN has been reported for several pathogens, including *Francisella*, *Listeria* and influenza virus. Over 40 genes known to be involved in regulating apoptosis are ISGs, thus a role for IFN-induced immune cell death in host susceptibility to plague will require the study of potentially many genes and their effects on *Y. pestis* pathogenesis.

*Y. pestis* joins a growing list of pathogens, such as *Listeria monocytogenes, Mycobacterium tuberculosis, Staphylococcus aureus* and *Francisella tularensis* that enhance virulence through IFNAR [10], [11], [12], [58], [59], [60], [61], [62]. Consistent in all of these models, the detrimental effect of IFN-I appears more pronounced under conditions of high pathogen burden. Yet, the molecular mechanism remains elusive. Because of the clear benefits of IFN-I on viral and cancer defense, use of this cytokine in humans is currently under investigation. Thus it is imperative that its potential side effects against bacterial infection be recognized and understood so they can be avoided while gaining the full therapeutic benefits of type I IFN.

## Materials and Methods

### Bacterial strains

Fully virulent *Y. pestis* KIM5^+^ was grown fresh from frozen stock by streaking for isolation onto heart infusion agar (HIA) plates supplemented with 0.005% Congo Red and 0.2% galactose to screen bacteria that retain the pigmentation locus [63]. For pneumonic plague challenge studies, a single pigmented colony was used to inoculate heart infusion broth (HIB) supplemented with 2.5 mM CaCl_2_ and grown 18–24 hrs at 37°C, 120 rpm. All handling of samples containing live *Y. pestis* KIM5^+^ was performed in a select agent authorized BSL3 facility under protocols approved by the University of Missouri Institutional Biosafety Committee. Non-pigmented *Y. pestis* strain KIM D27 was routinely grown fresh from frozen stock on HIA, followed by aerobic growth at 27°C in HIB overnight prior to use in experiments.

### Isolation of *Y. pestis* non-pigmented mutant (KIM5^−^)

A naturally occurring non-pigmented variant of the fully virulent *Y. pestis* KIM5^+^ strain was isolated following plating on Congo Red agar [63], [64]. Deletion of the pigmentation locus on the chromosome was verified by PCR as previously described prior to use in experiments [40].

### Animals

All animal procedures were in strict accordance with the Office of Laboratory Animal Welfare and the National Institutes of Health Guide for the Care and Use of Laboratory Animals and were approved by the University of Missouri Animal Care and Use Committee. Wild type C57BL/6 mice were commercially obtained from Charles River Laboratories (MA, USA). C57BL/6 mice were the inbred strain background of the *Irf3^−/−^*, *Irf7^−/−^*, and *Ifnar^−/−^* mice which were kind gifts of Drs Michael Diamond and Herbert Virgin [65], [66]. Mice were bred and raised at the University of Missouri barrier housing facilities. Male and female wild type and mutant mice, ranging from 15–30 g were used for challenge experiments. During challenge with fully virulent *Y. pestis* and the isogenic non-pigmented mutant, mice were maintained in select agent authorized animal biosafety level 3 facilities at the University of Missouri. All infected mice were monitored regularly by daily weighing and assignment of health scores. Animals that survived to the end of the 14 day observation period or were identified as moribund (defined by pronounced neurologic signs and severe weakness) were euthanized by CO_2_ asphyxiation followed by bilateral pneumothorax or cervical dislocation, methods approved by the American Veterinary Medical Association Guidelines on Euthanasia.

### Pulmonary plague challenge

Non-pigmented strains of *Y. pestis*, grown as described above, were diluted in sterile PBS to 1×10^6^ CFU/0.02 ml just prior to use in challenge experiments. Actual dose was determined by plating in triplicate on HIA. Unless otherwise indicated, for intranasal infections involving pgm^−^ *Y. pestis* strains, mice were given 50 µg FeCl_2_ by intraperitoneal injection just prior to challenge. For challenge with fully virulent *Y. pestis*, bacteria were diluted to 2×10^3^ CFU/0.02 ml sterile PBS. All animals were lightly anesthetized by isoflurane inhalation just prior to infection.

### Quantification of bacterial load in tissues

Immediately after euthanasia, blood was collected directly from the heart by cardiac puncture. Lungs, spleens and livers were removed, and half of each tissue was processed for bacterial load by homogenizing in 1 ml sterile PBS. Serial dilutions of homogenized tissues were then plated in triplicate onto HIA plates for quantification of bacterial titer (CFU/organ). Serum was collected from the blood following centrifugation and stored at −80°C until analyzed.

### Histopathologic evaluation of tissues

Approximately half of each tissue was placed in 10% formalin for 96 hrs. Lungs were first perfused with sterile PBS, removed and sectioned, then perfused with 10% formalin for histological analysis. Fixed tissues were embedded in paraffin, trimmed and stained with hematoxylin and eosin (H&E). For histologic scoring of tissues, slides were evaluated in a single blind fashion by a veterinarian with expertise in pathology. Lesions observed as well as the severity scores (0 to 3) were documented for lungs, liver and spleen. For enumeration of pyogranulomatous inflammatory foci in the liver, 10 fields per tissue were counted on each slide.

### Immunohistochemistry

Tissues that had been fixed in 10% formalin and paraffin-embedded as described above were sectioned for immunohistochemical analysis. Sections were stained with anti-rat F4/80 (AbD Serotec, Oxford, UK), monoclonal antibody NIMP-R14 (Santa Cruz Biotechnology, CA, USA) [67] or anti-rat Caspase-3 (Trevigen, MD, USA) and detection was achieved by secondary staining with biotinylated rabbit anti-rat IgG and HRP-streptavidin (DAKO, CA, USA). Staining and detection were carried out according to the manufacturer's guidelines. For scoring, ten fields were counted for positive caspase-3 staining on each slide and scored from 0–3 (0 = no caspase-3 staining, 1 = infrequent caspase-3 staining, 2 = moderately frequent caspase-3 staining, 3 = positive caspase-3 staining in majority of tissue).

### RNA isolation and Real Time PCR

Approximately half of the lung tissue was homogenized in RNAlater (Qiagen, CA, USA). RNA isolation was performed using RNeasy Mini Kit according to manufacturer's instructions (Qiagen, CA, USA). Total RNA was treated with Turbo DNase (Ambion, TX, USA) to remove genomic DNA contamination. First strand cDNA synthesis was carried out using MMLV-RT (Promega, WI, USA) on 2 µg of total RNA as per manufacturer's instructions. SYBR Green PCR master mix (Applied Biosystems, CA, USA) was used along with gene specific primers (Table S2) to detect the presence of amplified product. Results were analyzed using relative quantification on 7300 SDS software (Applied Biosystems, CA, USA). Data were normalized to the mouse gene *Ywhaz*, which is constitutively expressed with minimal change ([19], data not shown).

### Serum cytokine analysis

Blood from C57BL/6, *Irf3^−/−^* and *Ifnar^−/−^* mice was centrifuged and serum used for cytokine analysis with Premix 22-plex kit (Millipore, MA, USA) according to manufacturer's instructions and analyzed by Illuminex using IS 100 software (Qiagen, CA, USA). IL-2, IL-4 and IL-9 were undetectable in all samples and therefore they were removed from further analysis.

### Bone marrow-derived macrophage (BMDM) isolation

BMDMs were isolated from C57BL/6, *Irf3^−/−^* and *Ifnar^−/−^* mice essentially as previously described by culturing for 6 days in Dulbecco's modified Eagle's medium (DMEM) containing 20 ng/ml M-CSF (eBiosciences, CA, USA) in place of L cell media [68]. Twenty-four hours prior to infection, 1×10^6^ cells were seeded into 12-well plates with DMEM containing 20 ng/ml M-CSF, 10% fetal bovine serum (FBS). Where indicated, macrophages were pre-treated with 1 µg/ml anti-IFN-β or 500 µg/ml IFN-γ (Abcam, Cambridge, MA, USA) per well for 4 hrs prior to infection.

### Bone marrow neutrophil isolation

Primary bone marrow-derived neutrophils were isolated from femurs of wild type C57BL/6 or *Irf3^−/−^* mice. Neutrophils were enriched by separation on a three-layer Percoll (Sigma-Aldrich, St. Louis, MO) gradient. Purity of the isolated cells was assessed by microscopy at >95% neutrophils. Neutrophils were used in assays within 1 h of purification. Where indicated, neutrophils were pre-treated with PBS or anti-IFN-β 10 min prior to infection.

### Intracellular survival assay

Overnight cultures of *Y. pestis* KIM D27 were diluted 1∶20, incubated at 27°C for 3 hrs then shifted to 37°C for 1 hr. Bacteria were infected at a multiplicity of infection (MOI) of 10 and the plates were centrifuged at 86×g for 5 min before incubating at 37°C, 5%CO_2_. Following 30 min, cells were washed with sterile PBS and incubated in cell culture medium containing 40 µg/ml gentamicin and the infection continued for an additional 5.5 hrs. For bacterial titer, media was aspirated and wells were washed once with sterile PBS. Infected macrophages and neutrophils were lysed with 300 µl of 0.1% Triton X-100 in PBS, cells were then washed once with PBS, serially diluted and plated in triplicate on HIA for enumeration of colony forming units. To ensure that only intracellular bacteria had been enumerated, 10 µl of aspirated media was plated on HIA for each well and no bacterial growth was recovered in these samples (data not shown).

### Cytotoxicity assay

Lactate dehydrogenase (LDH) content was measured in the aspirated media collected from BMDMs infected as described above after 2 or 6 hrs of infection. Samples were run in triplicate using the CytoTox-One kit (Promega, WI, USA) and the manufacturer's instructions. Percent cytotoxicity was assessed by comparing the amount of LDH in the supernatant to that recovered from control BMDMs that were not infected following lysis by 0.1% triton X-100.

### Flow cytometry

Bone marrow cells from C57BL/6 and *Ifnar^−/−^* mice were isolated just prior to infection or on day 5 post-infection (dpi) from tibia and femurs using cold PBS. Cells were incubated with Fc receptor blocking solution (BioLegend, CA, USA) to eliminate non-specific binding for 10 min prior to fixing in 4% paraformaldehyde for 20 min. Cells were then washed 2× with cold PBS at 1200×g for 8 min and stained with FITC-conjugated anti-mouse Ly6G/6C antibody (BD Biosciences, CA, USA) for 30 min. Cells were washed 3× with cold PBS and analyzed on MoFlo XDP using Summit software (Beckman Coulter, UT, USA).

### Statistical analysis

Data from all trials were analyzed for statistical significance. Statistical analyses were performed using R (Gehan Wilcoxan, Kruskal Wallis) or GraphPad prism (Wilcoxan match paired, Student's t test, ANOVA, Mantel Cox) software [69].

## Supporting Information

Figure S1 **Increased GR-1^+^F4/80^+^ inflammatory foci in** ***Ifnar^−/−^*** **mice correlates with bacterial clearance.** Wild type C57BL/6 and *Ifnar^−/−^* mice were challenged by intranasal infection of 1×10^6^ CFU *Y. pestis* KIM D27 after pre-treatment with 50 µg Fe^+2^. Formalin fixed livers (left panels) and spleens (right panels) were prepared from wild type C57BL/6 (bottom) or *Ifnar^−/−^* (top) mice on day 5 post-infection, followed by sectioning and immunohistochemistry staining with anti-F4/80 (A) or anti-NIMP (B). Images shown are representative of two independent experiments, n = 6 per group, and were taken from mice from which bacteria could be recovered from both tissues. Scale bar indicates 50 µm.
(TIF)

Figure S2 **Increase in sensitivity of** ***Irf3^−/−^*** **mice occurs in the absence of pre-treatment with iron.** Groups of five wild type C57BL/6, *Irf3^−/−^*, and *Irf7^−/−^* mice were challenged by intranasal infection of 1×10^6^ CFU *Y. pestis* KIM D27 and monitored for survival over 14 days (n = 5 mice per group, single trial).
(TIF)

Figure S3 **IRF-7 is dispensable for host defense against** ***Y. pestis*** **.** Wild type C57BL/6 and *Irf7^−/−^* mice were challenged by intranasal infection of *Y. pestis* KIM D27. On days 2, 3 and 4 post-infection, lungs (circle), liver (triangle), and spleen (diamond) were harvested, homogenized in sterile PBS and plated to enumerate bacterial load per tissue. Open shapes are wild type and black shapes are *Irf7^−/−^*; data were collected in two independent experiments, each with 3 mice per group.
(TIF)

Figure S4 **Increased inflammation develops in the lungs of** ***Irf3^−/−^*** **mice.** Groups of three wild type C57BL/6 and *Irf3^−/−^* mice were challenged by intranasal infection of *Y. pestis* KIM D27. On day 3 post-infection, lungs were harvested, fixed in 10% formalin and analyzed by histochemistry; wild type (left), *Irf3^−/^*.(right). Scale bar indicates 100 µm. Images are representative of 6 mice.
(TIF)

Figure S5 ***Irf7^−/−^*** **mice develop inflammatory foci in the liver with little tissue necrosis on day 3 following pulmonary infection.** Groups of three *Irf7^−/−^* mice were challenged by intranasal infection of 1×10^6^ CFU *Y. pestis* KIM D27. On day 3 post-infection, animals were euthanized, tissues collected and fixed in 10% formalin. Hematoxylin and eosin (H&E) staining for lungs (top panels), liver (bottom left) and spleen (bottom right) of *Irf7^−/−^* mice. Histology for wild type mice on day 3 post-infection was shown in Figure 3. Scale bar indicates 100 µm. Images are representative of 6 mice.
(TIF)

Figure S6 **Increased apoptosis in phagocytic cells of** ***Irf3^−/−^*** **mice on day 3 correlates with accelerated disease progression.** Formalin fixed livers (A–F) and spleens (G–I) tissues collected from wild type C57BL/6 and *Irf3^−/−^* mice on days 2 (A, C) and 3 (B, D, F–I) post-infection (see figure 3) were analyzed by immunohistochemistry with anti-F4/80 (A–B, G), NIMP R14 (C–D, H), and anti-caspase-3 (F, I). For each panel of stains, wild type tissues are on the left, *Irf3^−/−^* tissues are on the right. Scale bar indicates 50 µm. Images are representative of 6 mice per group, collected in two independent experiments. (E) Day 3 foci of inflammation containing intact neutrophils in the liver were quantified by counting in 10 non-overlapping fields and the mean number counted from all fields was determined (n = 6 mice per group). (J) Mean severity scoring (0–3, with 3 indicating positive stain on the majority of the tissue) for caspase-3^+^ staining in lungs, liver and spleen on day 3 (n = 6 mice per group); *P<0.05 between wild type and *Irf3^−/−^* tissue, analyzed by unpaired Student's t-test.
(TIF)

Figure S7 **Increase in sensitivity of** ***Irf3^−/−^*** **mice is dependent on the absence of the** ***Y. pestis*** **pigmentation locus.** Male and female wild type C57BL/6 (n = 5) and *Irf3^−/−^* mice (n = 6) were challenged by intranasal infection of 1×10^6^ CFU *Y. pestis* KIM5^−^ and monitored for 14 days.
(TIF)

Table S1 **Expression of inflammatory molecules following pulmonary infection by** ***Y. pestis*** **KIM D27.**
(DOCX)

Table S2 **List of primers used for quantitative RT-PCR in this study.**
(DOCX)
