# Supplementary figures and images for: Opposing Roles for Interferon Regulatory Factor-3 (IRF-3) and Type I Interferon Signaling during Plague
Source: PLoS Pathog. 2012 Jul 26;8(7):e1002817. doi: 10.1371/journal.ppat.1002817 (PMC3406097; doi:10.1371/journal.ppat.1002817)

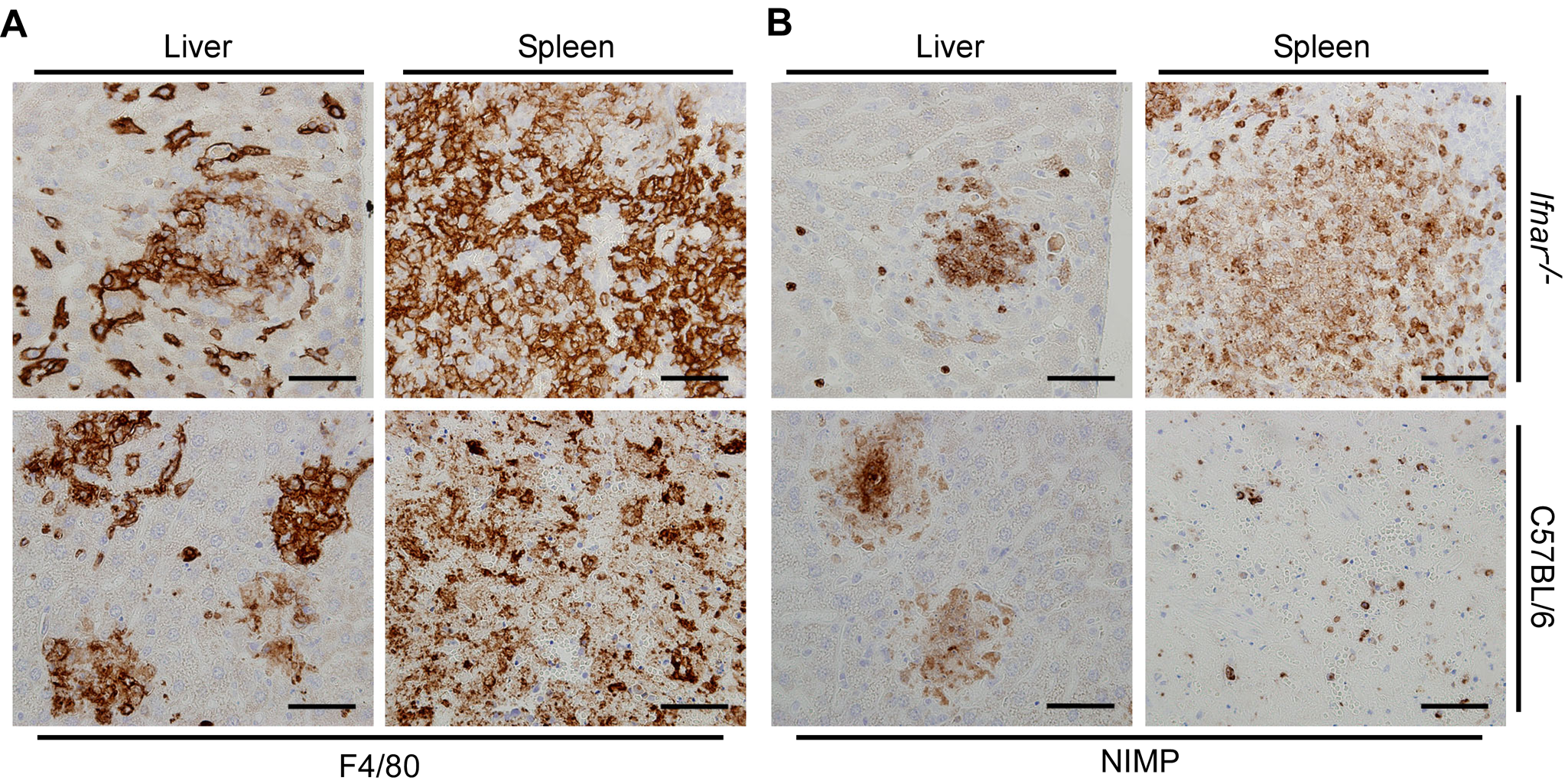

Supplement: Figure S1 — Increased GR-1+F4/80+ inflammatory foci in Ifnar−/− mice correlates with bacterial clearance. Wild type C57BL/6 and Ifnar−/− mice were challenged by intranasal infection of 1×10^6^ CFU Y. pestis KIM D27 after pre-treatment with 50 µg Fe+2. Formalin fixed livers (left panels) and spleens (right panels) were prepared from wild type C57BL/6 (bottom) or Ifnar−/− (top) mice on day 5 post-infection, followed by sectioning and immunohistochemistry staining with anti-F4/80 (A) or anti-NIMP (B). Images shown are representative of two independent experiments, n = 6 per group, and were taken from mice from which bacteria could be recovered from both tissues. Scale bar indicates 50 µm. (TIF) [file ppat.1002817.s001.tif]

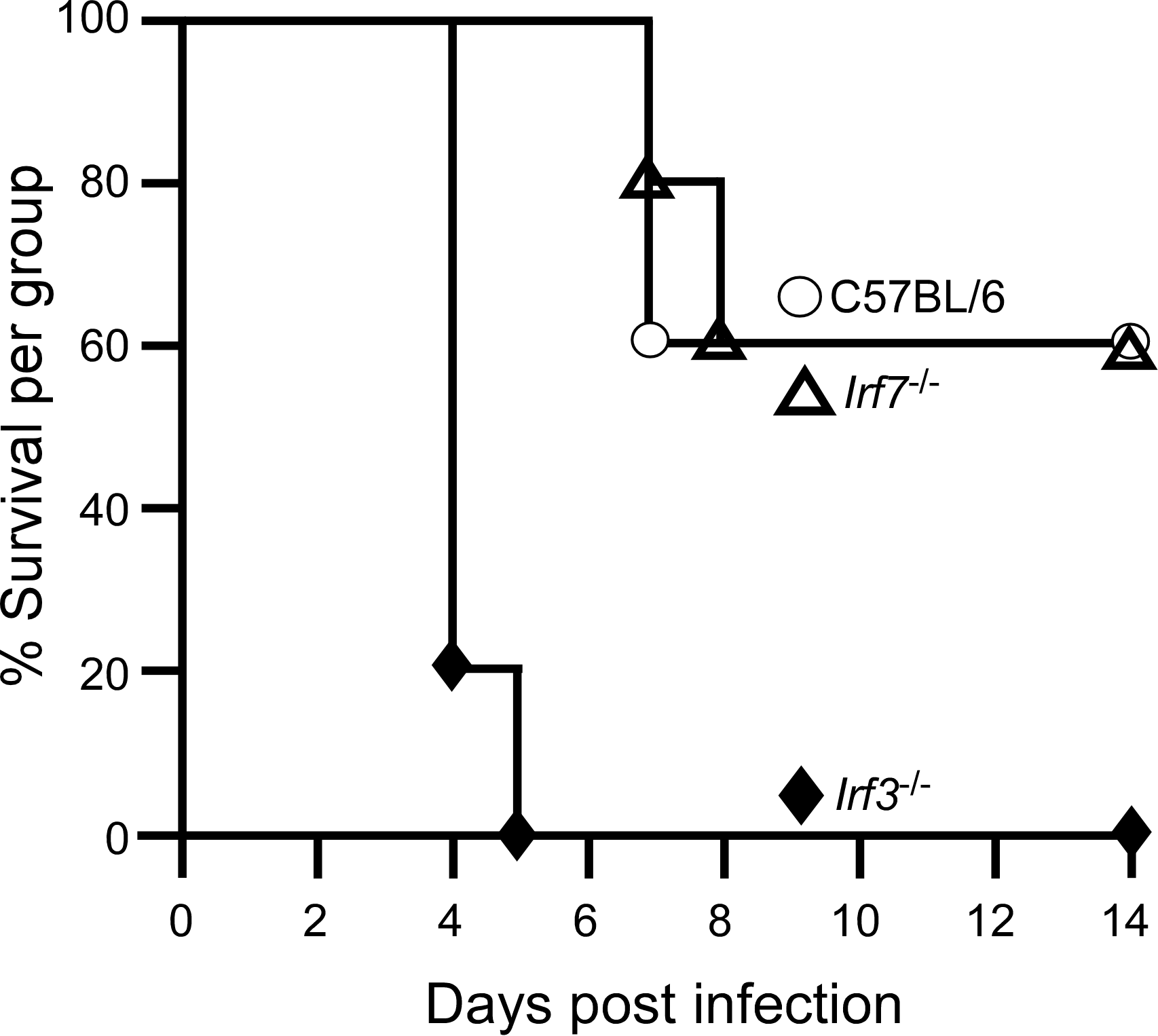

Supplement: Figure S2 — Increase in sensitivity of Irf3−/− mice occurs in the absence of pre-treatment with iron. Groups of five wild type C57BL/6, Irf3−/−, and Irf7−/− mice were challenged by intranasal infection of 1×10^6^ CFU Y. pestis KIM D27 and monitored for survival over 14 days (n = 5 mice per group, single trial). (TIF) [file ppat.1002817.s002.tif]

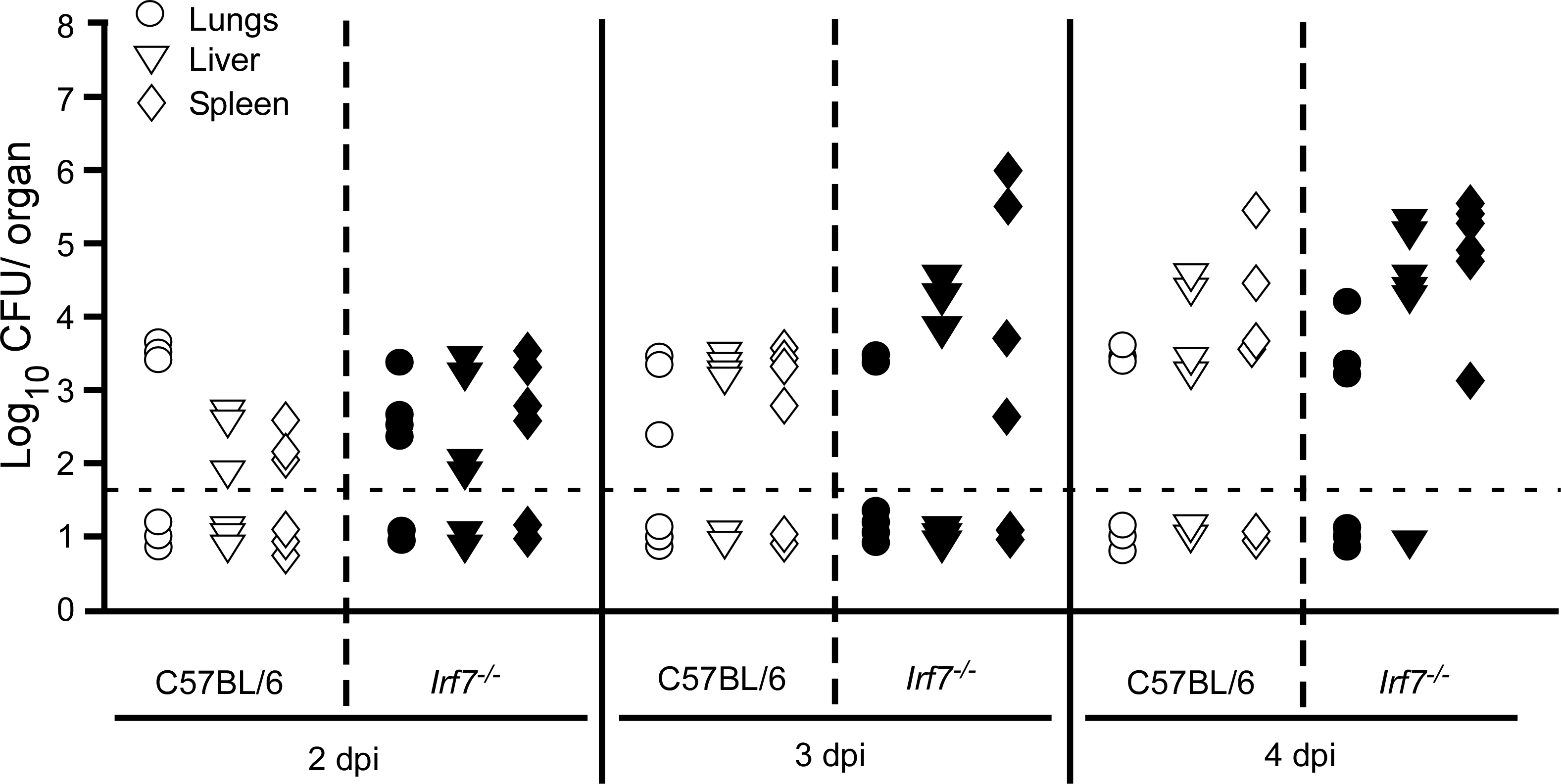

Supplement: Figure S3 — IRF-7 is dispensable for host defense against Y. pestis . Wild type C57BL/6 and Irf7−/− mice were challenged by intranasal infection of Y. pestis KIM D27. On days 2, 3 and 4 post-infection, lungs (circle), liver (triangle), and spleen (diamond) were harvested, homogenized in sterile PBS and plated to enumerate bacterial load per tissue. Open shapes are wild type and black shapes are Irf7−/−; data were collected in two independent experiments, each with 3 mice per group. (TIF) [file ppat.1002817.s003.tif]

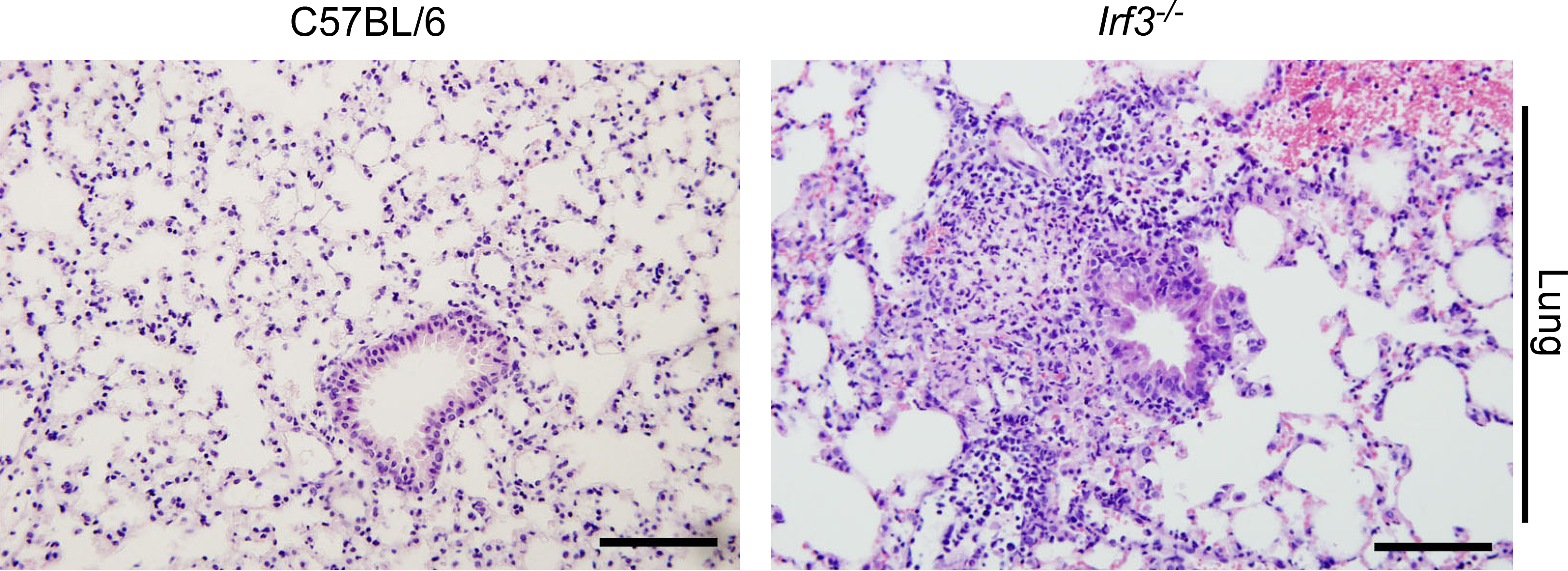

Supplement: Figure S4 — Increased inflammation develops in the lungs of Irf3−/− mice. Groups of three wild type C57BL/6 and Irf3−/− mice were challenged by intranasal infection of Y. pestis KIM D27. On day 3 post-infection, lungs were harvested, fixed in 10% formalin and analyzed by histochemistry; wild type (left), Irf3−/.(right). Scale bar indicates 100 µm. Images are representative of 6 mice. (TIF) [file ppat.1002817.s004.tif]

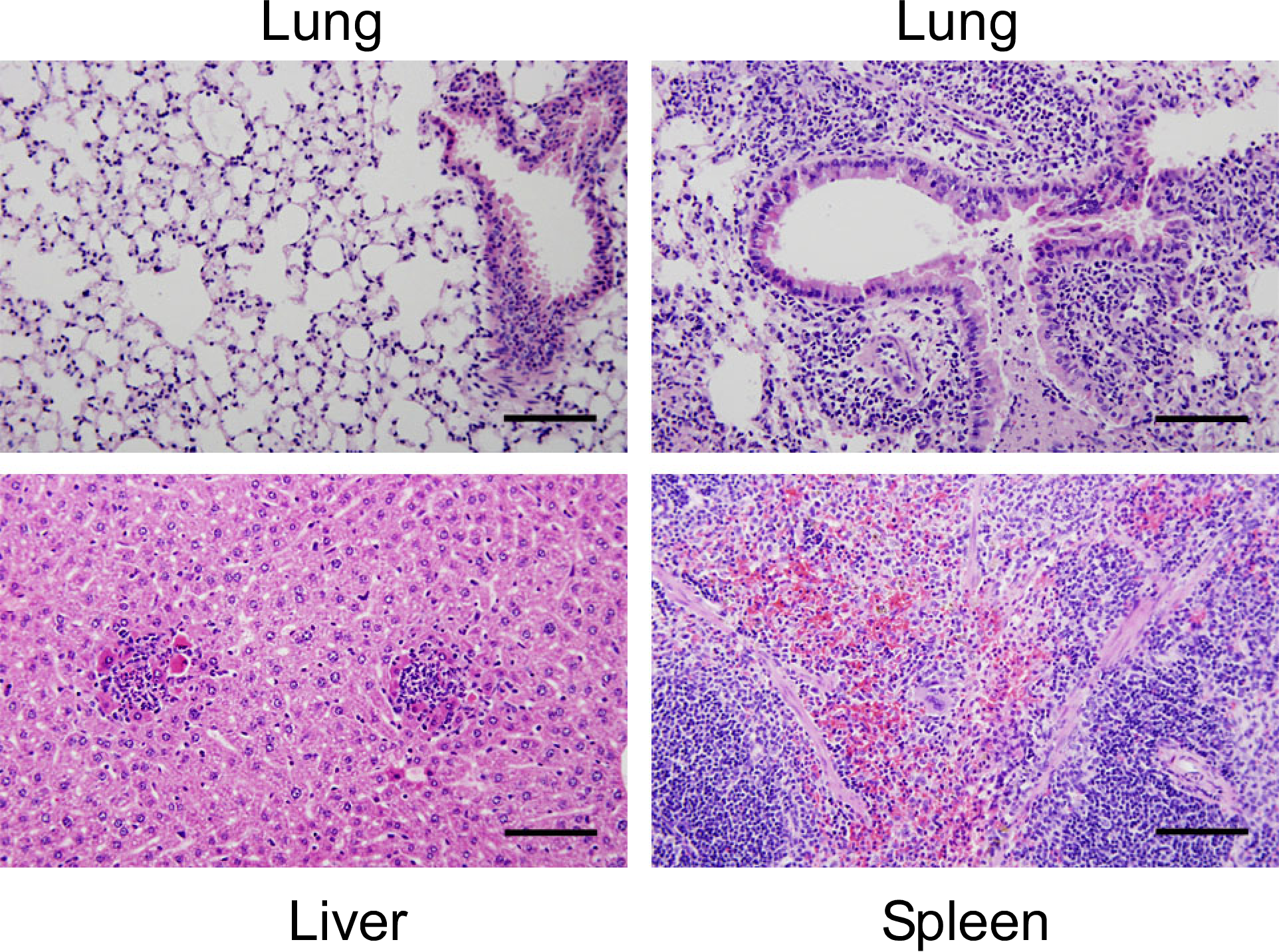

Supplement: Figure S5 — Irf7−/− mice develop inflammatory foci in the liver with little tissue necrosis on day 3 following pulmonary infection. Groups of three Irf7−/− mice were challenged by intranasal infection of 1×10^6^ CFU Y. pestis KIM D27. On day 3 post-infection, animals were euthanized, tissues collected and fixed in 10% formalin. Hematoxylin and eosin (H&E) staining for lungs (top panels), liver (bottom left) and spleen (bottom right) of Irf7−/− mice. Histology for wild type mice on day 3 post-infection was shown in Figure 3. Scale bar indicates 100 µm. Images are representative of 6 mice. (TIF) [file ppat.1002817.s005.tif]

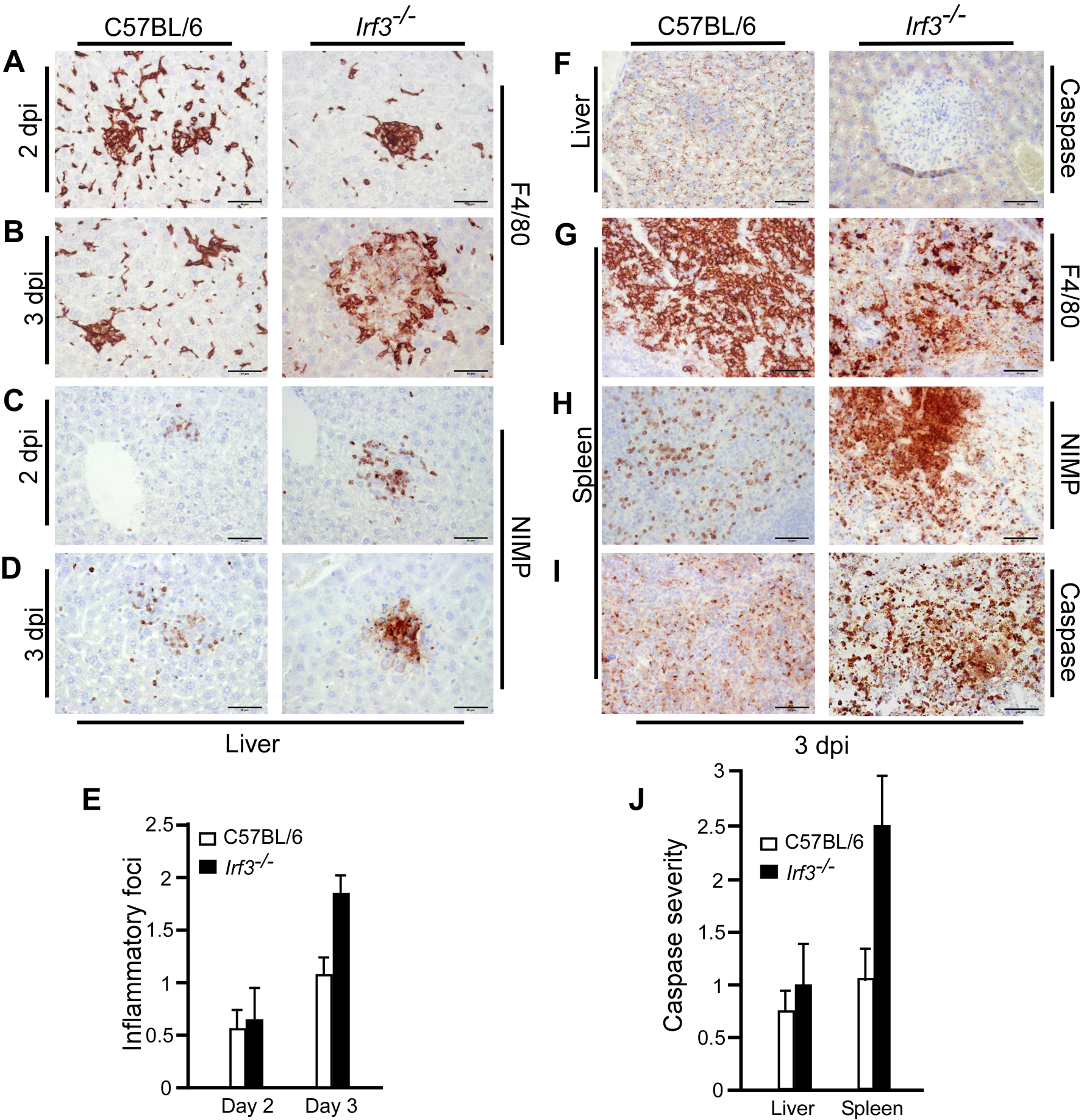

Supplement: Figure S6 — Increased apoptosis in phagocytic cells of Irf3−/− mice on day 3 correlates with accelerated disease progression. Formalin fixed livers (A–F) and spleens (G–I) tissues collected from wild type C57BL/6 and Irf3−/− mice on days 2 (A, C) and 3 (B, D, F–I) post-infection (see figure 3) were analyzed by immunohistochemistry with anti-F4/80 (A–B, G), NIMP R14 (C–D, H), and anti-caspase-3 (F, I). For each panel of stains, wild type tissues are on the left, Irf3−/− tissues are on the right. Scale bar indicates 50 µm. Images are representative of 6 mice per group, collected in two independent experiments. (E) Day 3 foci of inflammation containing intact neutrophils in the liver were quantified by counting in 10 non-overlapping fields and the mean number counted from all fields was determined (n = 6 mice per group). (J) Mean severity scoring (0–3, with 3 indicating positive stain on the majority of the tissue) for caspase-3+ staining in lungs, liver and spleen on day 3 (n = 6 mice per group); *P<0.05 between wild type and Irf3−/− tissue, analyzed by unpaired Student's t-test. (TIF) [file ppat.1002817.s006.tif]

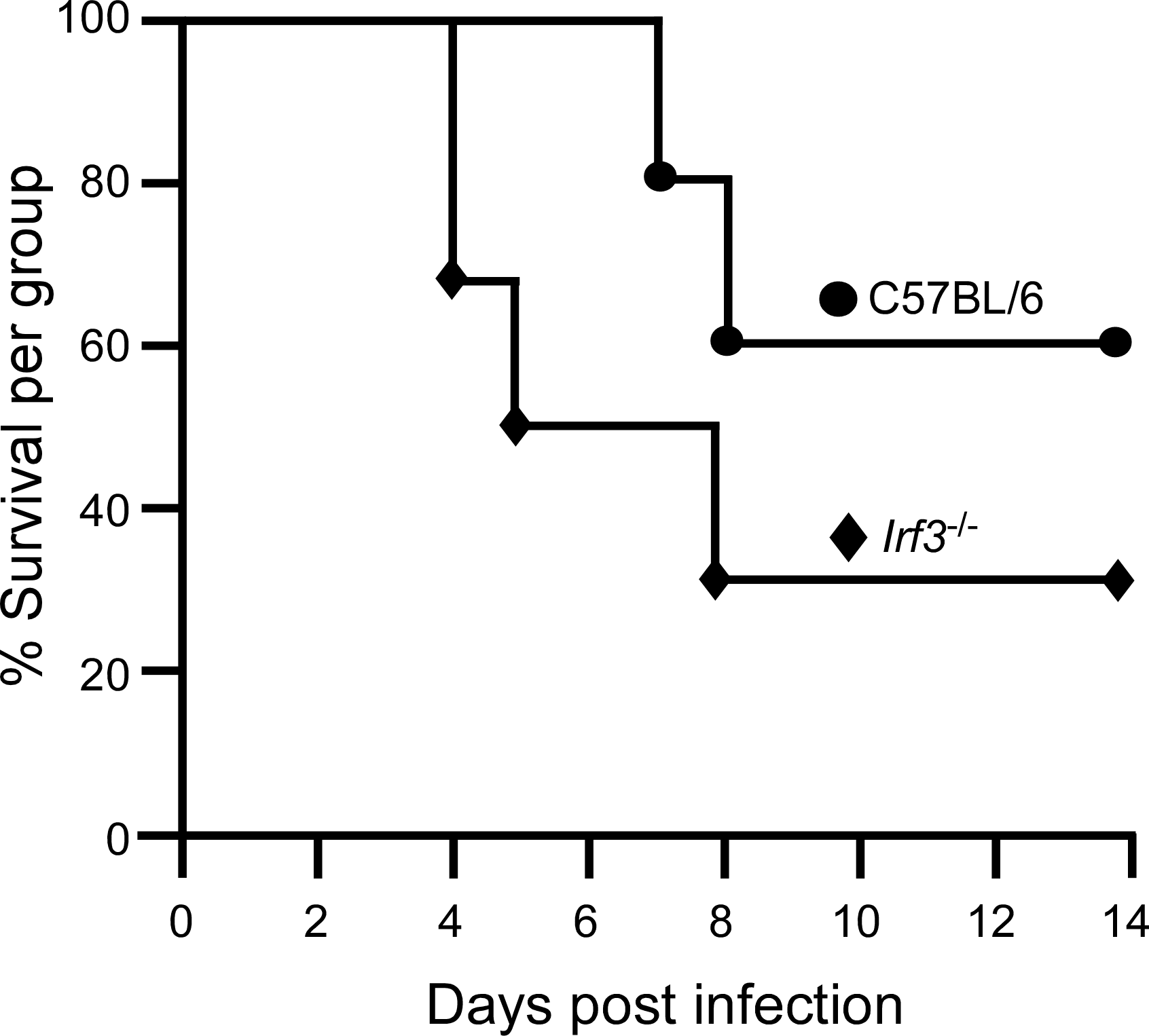

Supplement: Figure S7 — Increase in sensitivity of Irf3−/− mice is dependent on the absence of the Y. pestis pigmentation locus. Male and female wild type C57BL/6 (n = 5) and Irf3−/− mice (n = 6) were challenged by intranasal infection of 1×10^6^ CFU Y. pestis KIM5− and monitored for 14 days. (TIF) [file ppat.1002817.s007.tif]
