# Supplementary material for: Opposing Roles for Interferon Regulatory Factor-3 (IRF-3) and Type I Interferon Signaling during Plague
Source: PLoS Pathog. 2012 Jul 26;8(7):e1002817. doi: 10.1371/journal.ppat.1002817 (PMC3406097; doi:10.1371/journal.ppat.1002817)
Supplement: Table S1 — Expression of inflammatory molecules following pulmonary infection by Y. pestis KIM D27. (DOCX) [file ppat.1002817.s008.docx]

**Table S1.**

| **Gene** | **2 dpi^A^** | **4 dpi** | **7 dpi** |
| --- | --- | --- | --- |
| *Mcp1/Ccl2* | 35.41 ± 4.33* | 37.36 ± 14.45 | 375.89 ± 360.94* |
| *Mx1*  *IFNα1/6*  *IFNα8* | 23.69 ± 20.43*  1.07 ± 0.85  3.15 ± 2.86 | 7.89 ± 1.96*  1.16 ± 0.98  0.36 ± 0.30 | 4.11 ± 0.37*  1.55 ± 1.6  0.56 ± 0.55 |
| *Stat2* | 9.91 ± 8.02* | 6.34 ± 5.49* | 5.46 ± 1.54* |
| *Socs1* | 8.40 *±* 6.07* | 5.12 ± 4.33 | 10.64 ± 1.91* |

A: dpi: days post-infection

* p<0.05 compared to not infected.
