# Supplementary material for: Opposing Roles for Interferon Regulatory Factor-3 (IRF-3) and Type I Interferon Signaling during Plague
Source: PLoS Pathog. 2012 Jul 26;8(7):e1002817. doi: 10.1371/journal.ppat.1002817 (PMC3406097; doi:10.1371/journal.ppat.1002817)
Supplement: Table S2 — List of primers used for quantitative RT-PCR in this study. (DOCX) [file ppat.1002817.s009.docx]

**Table S2.**

| **Gene** | **Primer Pairs** | **Sequence (5’ – 3’)** |
| --- | --- | --- |
| *Irf*3 | *Irf*3F | ctgaccagtacaaggcctac |
|  | *Irf*3R | ggcataaactgctttattgg |
| *Ifnα1/6* | *Ifnα1*  *Ifnα1* | acctgctctctaggatgtga  tcctcatttgtaccaggagt |
| *Ifnα4* | *Ifnα4*  *Ifnα4* | gaagcatgtgtgatacagga  tggaagtatttcctcacagc |
| *Ifnα8* | *Ifnα8*  *Ifnα8* | gaggacatacttccacagga  ctccagacttctgctctgac |
| *IP10* | *IP10F*  *IP10R* | ccatagggaagcttgaaatc  tcagacatctctgctcatca |
| *Ifnβ* | *Ifnβ*F | caagatccctatggagatga |
|  | *Ifnβ*R | aagaaagacattctggagca |
| *Ifnγ* | *Ifnγ*F | ctgatgggaggagatgtcta |
|  | *Ifnγ*R | agcctgttactacctgacaca |
| *Tnfα* | *Tnfα*F | ccccaaagggatgagaagtt |
|  | *Tnfα*R | cacttggtggtttgcctacga |
| *Mcp1/Ccl2* | *Mcp*1/*Ccl*2F | ggaaccaaatgagatcagaa |
|  | *Mcp*1/*Ccl*2R | acttcacattcaaaggtgct |
| *Mx1* | *Mx1*F | catgggtcctttcatgtatt |
|  | *Mx1*R | tgaaatgatgtcttgctgaa |
| *Stat2* | *Stat2*F | tgtaagtccctggttctctg |
|  | *Stat2*R | gtacgaaggacttcaggaca |
| *Socs1* | *Socs1*F | gttgtagcagcttgtgtctg |
|  | *Socs1*R | tggtttgtgcaaagatactg |
| *Ywhaz* | *ywhF* | cacagcctcccctcatcct |
|  | *ywhR* | gggagacggtgacagaccat |
